# Amaryllidaceae Alkaloids and Isoquinoline Alkaloids: A Perspective on Historical Approaches to Pathway Elucidation

**DOI:** 10.3390/plants15172553

**Published:** 2026-08-22

**Authors:** Mateo Peña-Morales, Jaime David Vega-Páez, Natalie Cortes, Edison Osorio, Paola A. Caicedo, Alvaro Barrera-Ocampo, Oscar Álvarez, Andrés Fernando Gonzáles Barrios, María Francisca Villegas-Torres

**Affiliations:** 1 Grupo Natura, Facultad Barberi de Ingeniería, Diseño y Ciencias Aplicadas, Universidad ICESI, Cali 760031, Colombia; mapena@icesi.edu.co (M.P.-M.); pacaicedo@icesi.edu.co (P.A.C.); aabarrera@icesi.edu.co (A.B.-O.); 2 Centro de Investigaciones Microbiológicas (CIMIC), Department of Biological Sciences, Universidad de los Andes, Bogotá 111711, Colombia; jd.vega1754@uniandes.edu.co; 3 Facultad de Ciencias Naturales y Matemáticas, Universidad de Ibagué, Carrera, 22, Calle 67, Ibagué 730002, Colombia; charlotte08@gmail.com; 4 Grupo de Ciencias Básicas Aplicadas (CBATA), Departamento de Ciencias Básicas y Áreas Comunes, Tecnológico de Antioquia-Institución Universitaria, Medellín 050034, Colombia; edison.osorio@tdea.edu.co; 5 Grupo de Diseño de Productos y Procesos (GDPP), Department of Chemical and Food Engineering, Universidad de los Andes, Bogotá 111711, Colombia; oalvarez@uniandes.edu.co (O.Á.); andgonza@uniandes.edu.co (A.F.G.B.)

**Keywords:** isoquinoline alkaloids, Amaryllidaceae alkaloids, benzylisoquinoline alkaloids, alkaloid biosynthesis, transcriptomics, functional validation, CYP96T, norbelladine, 3,4-dihydroxybenzaldehyde, synthetic biology

## Abstract

Alkaloid biosynthesis is a central topic in plant specialized metabolism because many alkaloids have ecological, pharmacological, and biotechnological relevance. Isoquinoline alkaloids (IAs) and Amaryllidaceae alkaloids (AAs) are both connected to aromatic amino acid metabolism, but they differ in taxonomic distribution, scaffold-forming chemistry, pathway resolution, and biotechnological development. This review compares the historical and methodological trajectories that have shaped IA and AA pathway elucidation, from compound isolation, radiotracer experiments, and biochemical inference to transcriptomics, metabolomics, functional enzymology, isotope-guided active-tissue identification, regulatory studies, and heterologous pathway reconstruction. In IAs, especially benzylisoquinoline alkaloids, broad genomic and transcriptomic resources have supported candidate gene discovery and functional characterization of several branches, including morphinan, protoberberine, benzophenanthridine, and aporphine-related pathways. In contrast, AA biosynthesis has advanced more recently through function-driven approaches that clarified key steps such as N4OMT-mediated 4′-*O*-methylation, NBS/NR-mediated norbelladine formation, CYP96T-dependent regioselective oxidative coupling, and transient reconstruction of major scaffold-forming branches. Remaining gaps include the unresolved enzymatic formation of 3,4-dihydroxybenzaldehyde in AAs and incomplete functional validation across less-studied IA scaffold classes. By integrating biochemical logic, omics-guided discovery, enzyme evolution, tissue specificity, regulation, and synthetic biology, this review identifies priorities for future alkaloid pathway discovery and sustainable production.

## 1. Introduction

Specialized plant metabolites (SPMs) are chemically diverse compounds that contribute to plant adaptation, defense, communication, and interactions with the environment. These molecules are of considerable interest because of their structural diversity and broad industrial, agricultural, and pharmacological applications [1]. SPMs arise from central metabolic pathways and are further diversified through enzymatic modifications such as decarboxylation, hydroxylation, methylation, oxidation, reduction, glycosylation, and acylation, among others [2,3]. Based on their chemical structures, SPMs are commonly classified as phenolics, alkaloids, terpenes, saponins, lipids, and glycosylated compounds [3].

Among these groups, alkaloids are nitrogen-containing natural products widely recognized for their ecological roles and bioactivities. In plants, they often contribute to defense against herbivores and pathogens, although alkaloid production is not restricted to plants and has also been reported in fungi, bacteria, and animals [4]. Alkaloids typically contain at least one basic nitrogen atom, frequently within a heterocyclic ring, and display a wide range of biological activities, including antiviral, antibacterial, anti-inflammatory, antineoplastic, herbicidal, insecticidal, and fungicidal effects. Traditionally, alkaloids have been classified according to the biosynthetic origin of their carbon skeleton and the position of the nitrogen atom as true alkaloids, protoalkaloids, or pseudoalkaloids. True alkaloids are amino acid-derived compounds that usually contain nitrogen within a heterocyclic ring; protoalkaloids are also amino acid-derived but contain nitrogen outside the heterocycle or lack a nitrogen-containing ring; and pseudoalkaloids have carbon skeletons not directly derived from amino acids, although nitrogen may be incorporated during biosynthesis through amination, transamination, or related reactions [5,6].

**Figure 1:**
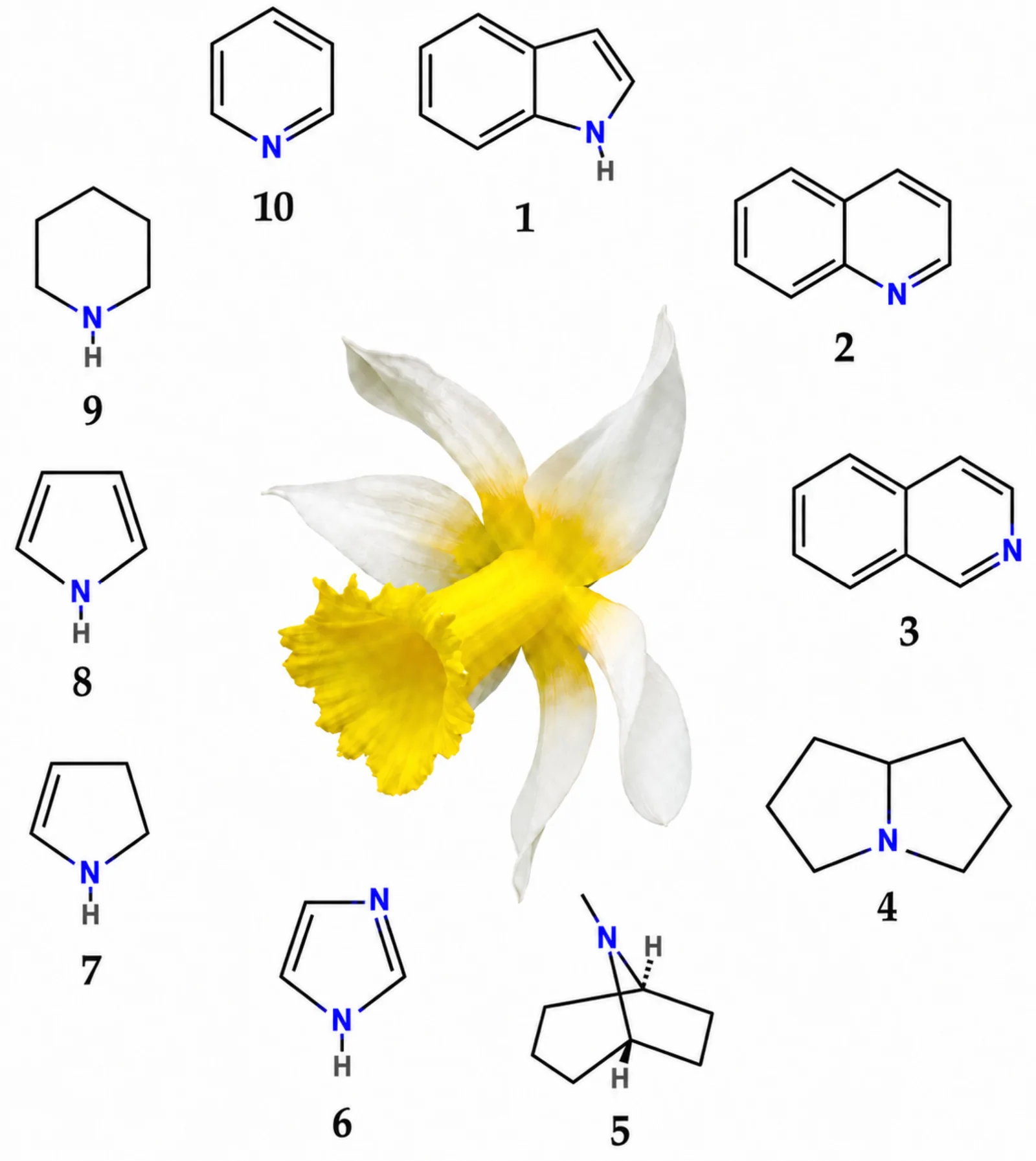
Representative skeletons of alkaloid diversity. (**1**) indole, (**2**) quinoline, (**3**) isoquinoline, (**4**) pyrrolizidine, (**5**) tropane, (**6**) imidazole, (**7**) pyrroline, (**8**) pyrrole, (**9**) piperidine, and (**10**) pyridine. The central flower illustration represents *Narcissus pseudonarcissus* (Amaryllidaceae) and is used only as a schematic visual element; it does not fully reflect the actual floral morphology of the species. Adapted from [1,8].

Alternatively, alkaloids can be classified according to defined chemical skeletons, such as indole, quinoline, isoquinoline, pyrrolizidine, tropane, imidazole, pyrroline, pyrrole, piperidine, and pyridine skeletons (Figure 1) [1]. However, biosynthetic classifications are particularly useful because they group alkaloids according to their biogenic precursors and pathway logic. This framework includes aromatic amino acid-derived alkaloids such as isoquinoline alkaloids (IAs) and Amaryllidaceae alkaloids (AAs), tryptophan-derived indole alkaloids, ornithine- or lysine-derived alkaloids, and alkaloids derived from polyamines, purines, terpenoids, or polyketide precursors [5,7]. A biosynthetic framework better captures evolutionary and mechanistic relationships among structurally diverse compounds, aligns with the organization of biosynthetic genes and enzyme families, and facilitates pathway engineering and genome-guided discovery. In contrast, purely structural classifications may separate biosynthetically related compounds and do not directly inform enzyme function or metabolic networks [1,4,7,8,9,10].

Isoquinoline alkaloids (IAs) are a broad structural class reported across multiple plant lineages. More than 4000 IAs have been described from at least ten plant families, including Papaveraceae, Berberidaceae, Rutaceae, Menispermaceae, Alangiaceae, Fabaceae, Ranunculaceae, Lauraceae, Annonaceae, and Fumariaceae [11]. In a systematic MS/MS study, Qing et al. examined 66 representative compounds belonging to 19 types of isoquinoline alkaloids, illustrating the structural breadth of this alkaloid class [11]. Broader surveys have reported IA-related compounds across approximately 28 plant families and 17 orders, illustrating the wide taxonomic distribution of this alkaloid class [8,9].

**Figure 2:**
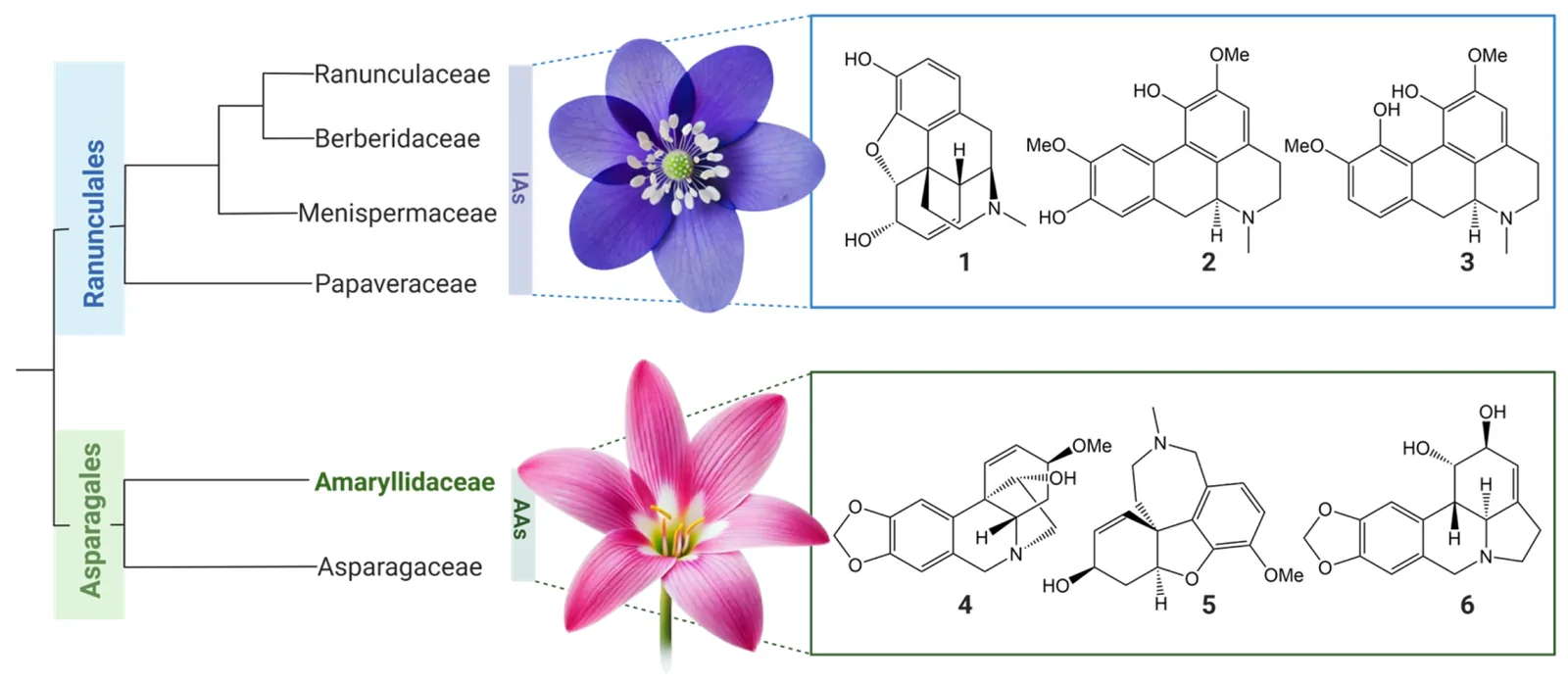
Representative phylogenetic comparison of IA- and AA-producing families. IAs have been reported across a broad taxonomic range, including multiple plant families and orders, whereas AAs are more lineage restricted. This simplified cladogram highlights selected IA-producing families within Ranunculales and representative AA-producing lineages within Asparagales. It is intended as an illustrative comparison rather than a comprehensive taxonomic survey. Representative IA structures are shown at the top: (**1**) morphine, (**2**) isoboldine, and (**3**) corytuberine. Representative AA structures are shown at the bottom: (**4**) haemanthamine, (**5**) galanthamine, and (**6**) lycorine. The flower illustrations, *Hepatica nobilis* for Ranunculaceae and *Zephyranthes carinata* for Amaryllidaceae, are used only as schematic examples of the corresponding plant families. The alkaloid structures shown are not exclusive to these species or to floral tissues.

In contrast, AAs are more taxonomically restricted and are mainly associated with Amaryllidaceae, particularly Amaryllidoideae, within Asparagales. Figure 2 therefore provides a representative, rather than comprehensive, phylogenetic comparison, highlighting selected IA-producing families within Ranunculales and representative AA-producing lineages within Asparagales. This phylogenetic contrast is accompanied by distinct structural outcomes: representative IA scaffolds include morphinan-, aporphine-, and protoberberine-related alkaloids, whereas representative AA scaffolds include haemanthamine-, galanthamine-, and lycorine-type alkaloids (Figure 2).

Both alkaloid groups are ultimately connected to shikimate-derived aromatic amino acid metabolism. In canonical benzylisoquinoline alkaloid (BIA) biosynthesis, L-tyrosine-derived intermediates provide both the dopamine unit and the aldehyde component, such as 4-hydroxyphenylacetaldehyde (4-HPAA), required for formation of the central benzylisoquinoline scaffold [7,12,13]. In AA biosynthesis, L-tyrosine provides tyramine, whereas L-phenylalanine is proposed to contribute to the aldehyde precursor 3,4-dihydroxybenzaldehyde (3,4-DHBA) through the phenylpropanoid pathway, although the complete enzymatic route to 3,4-DHBA remains unresolved [12].

Isoquinoline alkaloids should not be considered a single homogeneous biosynthetic group, but rather a broad chemical space comprising multiple structural types. Qing et al. examined 66 representative compounds belonging to 19 IA types, including benzyltetrahydroisoquinoline, aporphine, protoberberine, tetrahydroprotoberberine, protopine, benzophenanthridine, morphinan, phthalideisoquinoline, ipecac, bisbenzyltetrahydroisoquinoline, narciclasine, and related structural classes [11]. Although that study focused on fragmentation behavior rather than biosynthetic enzyme function, it illustrates the structural breadth of the IA family. This diversity is biosynthetically relevant because different IA scaffold types arise from distinct branch-point intermediates, oxidative coupling patterns, *O*- and *N*-methylation states, ring rearrangements, and lineage-specific enzyme recruitment. Therefore, throughout this review, IAs are treated as a broad comparative framework, while emphasis is placed on branches for which pathway elucidation has advanced most clearly, particularly benzylisoquinoline-derived morphinan, protoberberine, benzophenanthridine, and aporphine-related pathways.

The first committed step in benzylisoquinoline alkaloid (BIA) biosynthesis is the condensation of dopamine with 4-hydroxyphenylacetaldehyde (4-HPAA), catalyzed by norcoclaurine synthase (NCS), to produce (*S*)-norcoclaurine. This compound is the central entry intermediate of BIA biosynthesis and contains a tetrahydroisoquinoline scaffold with a nitrogen heterocycle and two phenolic rings [13]. The conversion of (*S*)-norcoclaurine to (*S*)-reticuline proceeds through a defined sequence of *O*- and *N*-methylation, P450-dependent hydroxylation, and additional tailoring reactions. Specifically, (*S*)-norcoclaurine undergoes 6-*O*-methylation by 6-*O*-methyltransferase (6OMT), followed by N-methylation by coclaurine *N*-methyltransferase (CNMT), cytochrome P450-dependent hydroxylation by CYP80B, and 4′-*O*-methylation by 4′OMT to yield (*S*)-reticuline [7,9,13].

**Figure 3:**
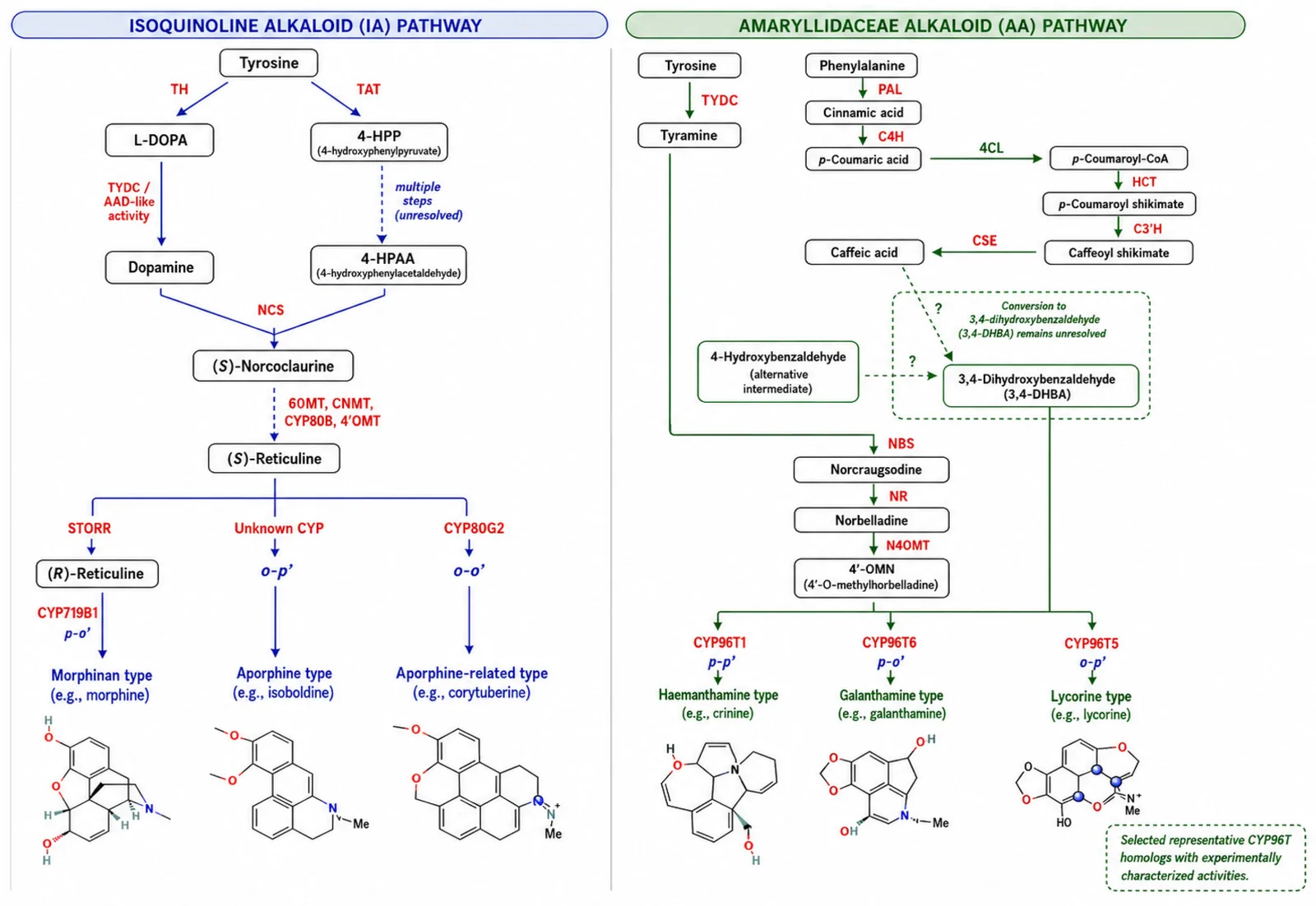
Comparative overview of IA and AA biosynthetic pathways. The IA pathway is highlighted in blue, whereas the AA pathway is shown in green. Enzymes involved in precursor supply or not restricted to a single alkaloid pathway are highlighted in red. Solid arrows indicate reactions catalyzed by a single characterized enzyme, whereas dashed arrows represent multi-step transformations or reactions for which the complete enzymatic sequence remains unresolved. Key intermediates are shown, and the carbon atoms involved in oxidative phenol-coupling reactions are color-coded to illustrate the formation of distinct scaffold types. The route from phenylpropanoid intermediates to 3,4-DHBA remains unresolved. Caffeic acid/caffeoyl derivatives and 4-hydroxybenzaldehyde have been proposed as possible intermediates, but the enzymatic steps leading directly to 3,4-DHBA have not been fully established. Norbelladine formation is depicted as a two-step process involving NBS-mediated imine formation followed by NR-mediated reduction. Selected CYP96T enzymes are shown as representative experimentally characterized examples of regioselective phenol-coupling catalysts in AA biosynthesis; however, CYP96T isoform numbering is not functionally equivalent across species, and the coupling reactions shown should therefore be interpreted according to the characterized homolog and species rather than by isoform name alone. The absence of the additional ortho-hydroxyl group in tyramine, compared with dopamine, helps explain why AA biosynthesis proceeds through linear norbelladine formation rather than Pictet–Spengler closure. Abbreviations: 4-HPP, 4-hydroxyphenylpyruvate; 4-HPAA, 4-hydroxyphenylacetaldehyde; L-DOPA, 3,4-dihydroxy-L-phenylalanine; 3,4-DHPA, 3,4-dihydroxyphenylacetaldehyde; 3,4-DHBA, 3,4-dihydroxybenzaldehyde; 4′-OMN, 4′-*O*-methylnorbelladine; TAT, tyrosine aminotransferase; TH, tyrosine hydroxylase; TYDC, tyrosine decarboxylase; AAD-like activity, aromatic amino acid decarboxylase-like activity; PAL, phenylalanine ammonia-lyase; C4H, trans-cinnamate 4-monooxygenase; 4CL, 4-coumarate-CoA ligase; C3H, 4-coumarate 3-hydroxylase; HCT, hydroxycinnamoyl transferase; C3′H, *p*-coumaroyl shikimate 3′-hydroxylase; CSE, caffeoyl shikimate esterase; NBS, norbelladine synthase; NR, norcraugsodine/noroxomaritidine reductase; NCS, norcoclaurine synthase.

Figure 3 presents a partial comparison of IA and AA biosynthetic logic, focusing on precursor origin, early scaffold-forming reactions, and oxidative phenol-coupling steps that generate major scaffold types. It is not intended to represent complete downstream pathways for all IA and AA classes. After (*S*)-reticuline formation, branch-specific enzyme systems direct BIA diversification. In morphinan biosynthesis, for example, the STORR fusion protein converts (*S*)-reticuline to 1,2-dehydroreticuline through its P450 module and then to (*R*)-reticuline through its oxidoreductase/reductase module. From (*R*)-reticuline, a CYP-mediated intramolecular C–C phenol-coupling reaction forms salutaridine, an early committed intermediate of the morphinan branch. Subsequent reductase-, acetyltransferase-, dioxygenase-, isomerase-, and demethylase-mediated steps lead to thebaine, codeine, and morphine. In parallel, (*S*)-reticuline and related branch-point intermediates can enter other IA branches through oxidative phenol coupling, methylenedioxy bridge formation, reductions, *O*- and *N*-methylations, and additional tailoring reactions, giving rise to protoberberine, aporphine, benzophenanthridine, protopine, and other scaffold types. Selected early BIA reactions and branch-forming transformations, together with the corresponding AA entry steps, are illustrated in Figure 3 [14,15,16,17,18,19,20].

Amaryllidaceae alkaloids (AAs) are aromatic amino acid-derived alkaloids found mainly in Amaryllidaceae, particularly within Amaryllidoideae, although related occurrences have also been reported in other Asparagales lineages (Figure 2) [21,22,23,24]. In contrast to canonical BIA pathways, AAs follow a distinct biosynthetic route based on two precursor units: tyramine, produced by decarboxylation of L-tyrosine, and 3,4-dihydroxybenzaldehyde (3,4-DHBA), which is proposed to originate from L-phenylalanine through the phenylpropanoid pathway. However, the complete enzymatic sequence leading to 3,4-DHBA remains unresolved. Proposed routes include caffeic acid- or caffeoyl-derived intermediates, as well as an alternative route through 4-hydroxybenzaldehyde. Recent characterization of *Leucojum aestivum* C4H/CYP73A and C3′H/CYP98A supports the involvement of upstream phenylpropanoid metabolism but does not establish the final enzymatic conversion to 3,4-DHBA [24,25].

Norbelladine formation proceeds through a two-step route in which NBS catalyzes the condensation of tyramine and 3,4-DHBA to form the imine intermediate norcraugsodine, followed by NR-mediated reduction to norbelladine [26,27,28,29]. Although NBS and NR can be assayed separately, recent evidence indicates that their combined activity more efficiently supports norbelladine formation, likely because NR reduces the unstable imine intermediate generated by NBS. Therefore, the current model favors a sequential NBS–NR route rather than a single-enzyme condensation/reduction step [26,27,29]. Norbelladine is then converted by norbelladine 4′-O-methyltransferase (N4OMT), which catalyzes 4′-*O*-methylation to form 4′-*O*-methylnorbelladine (4′-OMN), the central branch-point intermediate of AA biosynthesis [30].

From 4′-OMN, CYP96T-mediated oxidative coupling generates three major scaffold-forming routes: para–para′ coupling toward haemanthamine/crinine-type alkaloids, para–ortho′ coupling toward galanthamine-type alkaloids, and ortho–para′ coupling toward lycorine-type alkaloids. These scaffold classes include pharmacologically relevant compounds such as haemanthamine, crinine, galanthamine, lycorine, and montanine. Because these compounds differ in their reported biological activities, they are discussed here primarily as representatives of distinct AA biosynthetic branches rather than as a comprehensive pharmacological survey [31,32,33].

Recent experimental studies have substantially refined this model. Mehta et al. [32] used isotope-guided identification of biosynthetically active leaf-base tissues, RNA-seq/Iso-Seq, transient expression in *Nicotiana benthamiana*, and LC-MS/MS analyses to identify enzymes involved in the galanthamine, haemanthamine, and lycorine branches in *Narcissus* cv. Tête-à-Tête. Liu et al. [33] functionally characterized CYP96T1-like homologs from *Lycoris aurea*, showing that closely related enzymes can catalyze distinct para–para′ and para–ortho′ oxidative coupling reactions of 4′-OMN. Lamichhane et al. [34] further reconstituted key AA biosynthetic steps from *Leucojum aestivum* in *N. benthamiana*, including norbelladine formation, 4′-*O*-methylation, and CYP96T-mediated phenol-coupled products. Together, these findings demonstrate that AA scaffold diversification depends not simply on CYP96T isoform names, but on experimentally verified homologs, species context, and regioselective enzyme function [32,33,34].

Both IA and AA biosynthesis are rooted in shikimate-derived aromatic amino acid metabolism, which provides L-tyrosine and L-phenylalanine as key upstream precursors. In canonical BIA biosynthesis, L-tyrosine-derived intermediates provide both the dopamine unit and the aldehyde component required for formation of (S)-norcoclaurine. In AA biosynthesis, L-tyrosine contributes to tyramine, whereas L-phenylalanine is proposed to supply the catechol aldehyde precursor 3,4-dihydroxybenzaldehyde (3,4-DHBA) through the phenylpropanoid pathway. Thus, differences between IA and AA biosynthesis begin upstream of the alkaloid-specific pathway, at the level of aromatic amino acid allocation and precursor recruitment [12].

The comparison shown in Figure 3 highlights two distinct strategies for converting aromatic amino acid derivatives into complex alkaloid scaffolds. In BIAs, dopamine and 4-hydroxyphenylacetaldehyde are condensed by norcoclaurine synthase (NCS), a PR10/Bet v1-related enzyme, through a stereoselective Pictet–Spengler reaction to produce (*S*)-norcoclaurine, the central entry intermediate of BIA biosynthesis [13,35]. In plants, dopamine can arise from L-DOPA through TYDC or aromatic amino acid decarboxylase-like activity, whereas tyramine is formed by TYDC-mediated decarboxylation of L-tyrosine. These upstream decarboxylation reactions are therefore treated in Figure 3 as precursor-supply steps rather than as pathway-specific enzymatic steps.

In contrast, AA biosynthesis combines tyramine, derived from L-tyrosine, with 3,4-DHBA, proposed to originate from L-phenylalanine through the phenylpropanoid pathway. Norbelladine synthase (NBS), also related to the PR10/Bet v1 protein family, catalyzes imine formation from tyramine and 3,4-DHBA to produce norcraugsodine, which is subsequently reduced by NR to norbelladine [26,27,29]. This NBS/NR-mediated sequence defines the entry point into AA biosynthesis, although detailed kinetic characterization of this enzyme pair remains limited.

Thus, the first committed scaffold-forming steps differ substantially between the two pathways. BIA biosynthesis rapidly generates a cyclic tetrahydroisoquinoline scaffold through NCS-mediated Pictet–Spengler condensation, whereas AA biosynthesis first produces the linear secondary amine norbelladine, which must undergo 4′-*O*-methylation to form 4′-OMN before oxidative scaffold diversification. Despite these differences, both pathways converge mechanistically in their reliance on CYP-mediated oxidative transformations as major diversification steps.

In IAs, CYP80 and CYP719 enzymes participate in branch-specific oxidative reactions, including C–C phenol coupling and methylenedioxy bridge formation. For example, salutaridine synthase/CYP719B1 catalyzes intramolecular C–C phenol coupling of (*R*)-reticuline toward the morphinan branch, whereas CYP80G2 catalyzes intramolecular C–C phenol coupling of (*S*)-reticuline to produce (*S*)-corytuberine in the aporphine/magnoflorine-related branch [36,37]. In AAs, CYP96T homologs catalyze regioselective oxidative coupling of 4′-OMN, generating para–para′, para–ortho′, or ortho–para′ products associated with haemanthamine/crinine, galanthamine-, and lycorine-type scaffolds, respectively [31,32,33].

In this notation, the prime symbol (′) refers to the aromatic ring derived from tyramine, whereas the unprimed position refers to the aromatic ring derived from 3,4-DHBA. Thus, para–para′, para–ortho′, and ortho–para′ describe the relative positions on the two aromatic rings of 4′-OMN that participate in oxidative C–C bond formation. Therefore, although both IAs and AAs use oxidative phenol coupling to generate scaffold diversity, the enzymes, substrates, and regioselective outcomes differ between the two alkaloid groups.

This mechanistic comparison indicates that IA and AA biosynthesis should not be interpreted simply as parallel linear pathways, but as examples of how plant specialized metabolism can recruit related catalytic strategies to generate structurally distinct alkaloid families. These shared strategies include amine–aldehyde condensation, *O*- and *N*-methylation, reduction, hydroxylation, and oxidative phenol coupling, although the substrates, enzymes, and scaffold outcomes differ between the two alkaloid groups. Recent evidence from berberine biosynthesis further supports this view. In *Phellodendron amurense*, berberine formation appears to involve alternative enzymatic solutions, including *O*-methyltransferases with *N*-methylation activity and a cytochrome P450 replacing the classical FAD-dependent berberine bridge enzyme described in Ranunculales [38]. This example illustrates that similar alkaloid scaffolds can emerge through convergent or divergent enzymatic routes, emphasizing the need to combine structural chemistry, phylogeny, and functional validation when interpreting IA and AA pathway evolution.

Although AAs have been extensively isolated and chemically characterized from numerous Amaryllidaceae species, the molecular understanding of their biosynthesis remains less complete than that of several well-studied IA pathways. This gap is not due to a lack of chemical diversity, but rather to the limited availability of genomic resources, the restricted taxonomic scope of functional studies, and the difficulty of connecting transcriptomic candidates with experimentally validated enzymes. Most AA-related omics studies have focused on a few genera, mainly *Narcissus*, *Lycoris*, and *Leucojum*, and have often examined tissue specificity, developmental stages, elicitor responses, or metabolite accumulation patterns. These studies have been essential for candidate discovery, but recent advances show that transcriptomics alone is insufficient unless combined with metabolomics, isotope tracing, biochemical assays, protein–protein interaction studies, and heterologous pathway reconstruction.

The comparison between IAs and AAs provides a useful framework for examining how related biochemical themes can lead to distinct pathway architectures (Table 1). Although IAs have been studied for a longer period and generally have broader genomic and transcriptomic resources than AAs, this review does not treat both groups as equivalent pathways. Instead, it compares them as two alkaloid systems that share aromatic amino acid-derived precursors, amine–aldehyde chemistry, *O*- and *N*-methylation, reductions, hydroxylations, and oxidative phenol coupling, while differing in precursor combination, first committed scaffold-forming reaction, taxonomic distribution, pathway resolution, and biotechnological maturity. Three interpretive axes guide this comparison: scaffold assembly, represented by NCS-mediated Pictet–Spengler cyclization in IAs versus NBS/NR-mediated imine formation and reduction in AAs [13,26,27,29,35]; scaffold diversification, represented mainly by CYP80/CYP719-mediated transformations in IAs and CYP96T-mediated oxidative coupling in AAs [31,32,33,34,36,37]; and methodological development, from tracer-based pathway inference to omics-guided candidate discovery, functional validation, isotope-guided tissue selection, and heterologous pathway reconstruction [32,34,39,40,41,42]. This framework allows us to ask not only which genes have been identified, but also why some pathways have been resolved earlier than others, how taxonomic distribution constrains pathway discovery, and how enzyme recruitment and functional divergence shape alkaloid diversity.

## 2. Historical Landmarks in IA and AA Pathway Elucidation

The elucidation of alkaloid biosynthetic pathways has a long history that predates modern omics technologies. Morphine, for example, has been recognized for more than two centuries, following its isolation in 1805 by Friedrich Wilhelm Adam Sertürner, who referred to it as *Principium somniferum* [43]. By 1967, the morphine biosynthetic pathway had already been reviewed based on tracer experiments and analogous chemical transformations, proposing major steps from tyrosine to morphine through intermediates such as norlaudanosoline, reticuline, salutaridine, salutaridinol, thebaine, and codeine [39]. Although some of these early assignments were later revised, these studies established the conceptual framework for understanding morphinan biosynthesis before the advent of molecular and omics-based approaches.

In the case of IA biosynthesis, an early proposal in 1981 suggested norlaudanosoline as the first natural intermediate, with 3,4-dihydroxyphenylacetaldehyde (3,4-DHPA) as the aldehyde precursor, based on experiments using tritium-labeled dopamine and cell-free enzyme systems [44]. However, subsequent tracer experiments using carbon-labeled substrates demonstrated that the enzyme does not produce norlaudanosoline in plants. Instead, it catalyzes the formation of norcoclaurine from dopamine and 4-hydroxyphenylacetaldehyde, leading to its reassignment as norcoclaurine/norlaudanosoline synthase, now commonly referred to as norcoclaurine synthase (NCS) [45]. This correction illustrates how early pathway models were progressively refined as more precise biochemical evidence became available.

Beyond radiolabelling, gene expression studies became important tools for pathway elucidation. In 1995, in situ RNA hybridization showed differential and organ-dependent accumulation of tyrosine/dopa decarboxylase (TYDC) transcripts in opium poppy, suggesting coordinated regulation of specific alkaloid biosynthetic genes [46]. In 2001, recombinant DNA approaches, heterologous expression, and enzyme purification enabled the molecular characterization of salutaridinol 7-*O*-acetyltransferase, marking a transition from pathway mapping to gene-level enzymology in morphinan biosynthesis [16]. Later, immunofluorescence labeling and shotgun proteomics showed that morphine biosynthesis involves two specialized cell types, sieve elements and laticifers, adding spatial resolution to pathway understanding [47].

A major milestone was reached in 2015 with the identification of the STORR locus, which encodes a cytochrome P450–oxidoreductase fusion protein responsible for the conversion of (*S*)-reticuline to (*R*)-reticuline, a key step directing metabolism toward morphinan alkaloids [14]. This discovery clarified a long-standing gap in morphine biosynthesis and supported later efforts toward heterologous reconstruction of morphinan pathways. In 2019, neopinone isomerase was identified as the enzyme responsible for the conversion of neopinone to codeinone, a reaction previously assumed to occur spontaneously [17]. To date, several synthetic biology systems have been engineered to produce morphinan and related benzylisoquinoline alkaloids, and even feedback inhibition in engineered systems has begun to be investigated as a strategy to improve production [48,49,50]. Together, these findings show that IA pathway elucidation has evolved from tracer-based biochemical inference to gene discovery, spatial localization, enzyme characterization, and pathway engineering.

AAs also have a long chemical history. Lycorine, one of the first known Amaryllidaceae alkaloids, was isolated from *Narcissus pseudonarcissus* by A. W. Gerrard in 1878 during investigations into the poisonous properties of Amaryllidaceae plants [51]. Further studies on lycorine and related compounds were conducted in *Lycoris* species, with additional alkaloids described during the late nineteenth and early twentieth centuries. In 1957, Barton and Cohen proposed that the structural diversity of Amaryllidaceae alkaloids, including lycorine-, crinine-, and galanthamine-type compounds, could arise from oxidative phenol coupling of a common precursor [52]. This proposal anticipated the modern view that different regioselective phenol-coupling reactions of 4′-O-methylnorbelladine generate the major AA scaffold types.

Radiolabelling experiments were also central to early AA pathway elucidation. These studies helped establish that AA biosynthesis depends on two aromatic amino acid-derived precursor units: 3,4-dihydroxybenzaldehyde, proposed to originate from L-phenylalanine through the phenylpropanoid pathway, and tyramine, derived from L-tyrosine. The condensation of these two units leads to norbelladine, a central intermediate in AA biosynthesis [52]. A major advance came in 1998, when ^13^C-labeled 4′-O-methylnorbelladine was applied to *Leucojum aestivum* to investigate galanthamine biosynthesis, supporting its role as a key pathway intermediate [40]. Although these studies clarified the chemical logic of the pathway, the molecular basis of AA biosynthesis remained largely unexplored until the last decade.

The first AA-specific enzyme characterized at the molecular level was norbelladine 4′-*O*-methyltransferase (N4OMT), which catalyzes the 4′-*O*-methylation of norbelladine to produce 4′-*O*-methylnorbelladine (4′-OMN), the central branching intermediate for downstream phenol-coupling reactions [30]. Subsequently, norbelladine synthase (NBS) and norcraugsodine/noroxomaritidine reductase (NR) were characterized as enzymes acting before the phenol-coupling step, while CYP96T1 was identified as the cytochrome P450 responsible for the para–para′ oxidative coupling that leads to haemanthamine-type intermediates [26,28,31]. More recently, functional studies have expanded the experimentally supported AA biosynthetic pathway, particularly the branches downstream of 4′-OMN. In 2024, Mehta et al. identified CYP96T6 and CYP96T5 as enzymes involved in para–ortho′ and ortho–para′ coupling, respectively, and described additional enzymes required for galanthamine and haemanthamine biosynthesis, including SDR1, SDR2, CYP71DW1, ODD2, OMT1, NMT1, and AKR1 [32]. In parallel, Lamichhane et al. reported CYP96T enzymes from *Leucojum aestivum* capable of catalyzing regioselective phenol-coupling reactions, further supporting the central role of CYP96T diversification in AA scaffold formation [34].

**Figure 4:**
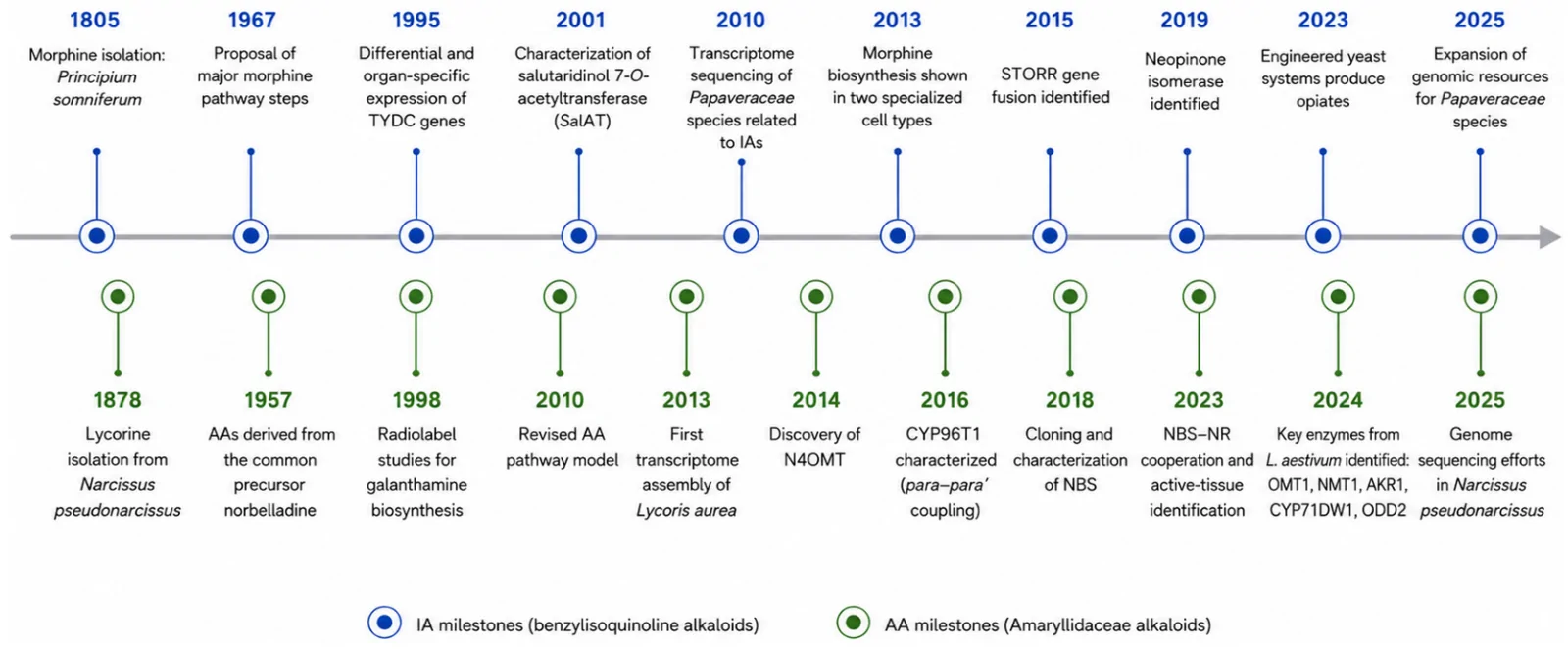
Historical landmarks in the elucidation of IA and AA biosynthetic pathways. The elucidation of IA and AA biosynthesis began with the isolation of emblematic compounds such as morphine and lycorine, followed by early pathway proposals based mainly on radiotracer feeding experiments. Over time, biochemical, molecular, and omics-based approaches enabled the identification of key enzymes, spatial and temporal characterization of pathway activity, and reconstruction of biosynthetic steps in heterologous systems. The timeline highlights the earlier transition of IA research toward gene-level, genomic, and biotechnological applications, whereas AA biosynthesis remained largely supported by chemical evidence until recent advances in transcriptomics, functional enzymology, isotope-guided tissue selection, CYP96T regioselectivity analysis, and pathway reconstruction. For the morphinan branch, the timeline summarizes the progressive elucidation of the sequence from (*S*)-reticuline to morphine, including the identification of STORR and neopinone isomerase.

Taken together, these historical milestones show that IA and AA pathway elucidation followed different temporal trajectories. IA research moved earlier from radiotracer-based pathway inference to enzyme purification, molecular cloning, spatial pathway localization, genomics, and synthetic biology [13,14,39,47,49,53]. In contrast, AA biosynthesis remained supported mainly by chemical and radiolabelling evidence for a longer period, with rapid molecular advances emerging more recently through N4OMT, CYP96T1, NBS, NR, isotope-guided active-tissue identification, and transient pathway reconstruction [26,27,28,29,30,31,32,33,34,40,52]. As summarized in Figure 4, this historical contrast provides the basis for comparing not only the pathways themselves, but also the methodologies that enabled their elucidation.

The milestones summarized in Figure 4 are not only chronological markers but also represent methodological transitions that changed the type of biological questions that could be addressed. Early isolation, chemical degradation, and radiotracer studies established precursor–product logic by identifying carbon flow and proposing central intermediates such as reticuline, norbelladine, and 4′-*O*-methylnorbelladine [39,40,52]. In morphinan biosynthesis, these approaches helped define a route from aromatic amino acid-derived precursors through dopamine, 4-hydroxyphenylacetaldehyde, (*S*)-norcoclaurine, and (*S*)-reticuline toward morphine-related intermediates. In its currently accepted form, the morphinan branch proceeds through STORR-mediated conversion of (*S*)-reticuline to (*R*)-reticuline, followed by transformation through salutaridine, salutaridinol, salutaridinol-7-*O*-acetate, thebaine, neopinone, codeinone, and codeine to yield morphine [14,17,48,49]. Thus, morphine biosynthesis illustrates how early biochemical inference was progressively converted into a gene- and enzyme-resolved pathway.

However, radiotracer and chemical approaches could not identify the genes, enzymes, cell types, or regulatory mechanisms responsible for pathway flux. The transition to molecular cloning, transcriptomics, proteomics, genomics, and heterologous reconstruction shifted the field from pathway inference to pathway testing. This transition occurred earlier in IAs, where the characterization of NCS, SalAT, cell-type-specific morphine biosynthesis, the STORR fusion protein, neopinone isomerase, and engineered yeast systems transformed morphinan biosynthesis into a reconstructable pathway [13,14,17,47,48,49,54]. In AAs, a comparable transition has occurred more recently through N4OMT, NBS, NR, CYP96T enzymes, isotope-guided tissue selection, CYP regioselectivity analysis, and transient reconstruction in *Nicotiana benthamiana*, moving the field from chemically inferred pathways toward experimentally validated and engineerable modules [26,27,28,29,30,31,32,33,34]. To complement the historical timeline and avoid presenting enzyme discovery as a simple list of names, Supplementary Table S1 summarizes representative functionally characterized enzymes in IA and AA biosynthesis according to year of discovery, source species, substrate, product or reaction, and experimental strategy used for functional assignment.

### 2.1. Omics-Guided Discovery and Functional Validation of IA Biosynthetic Genes

**Figure 5:**
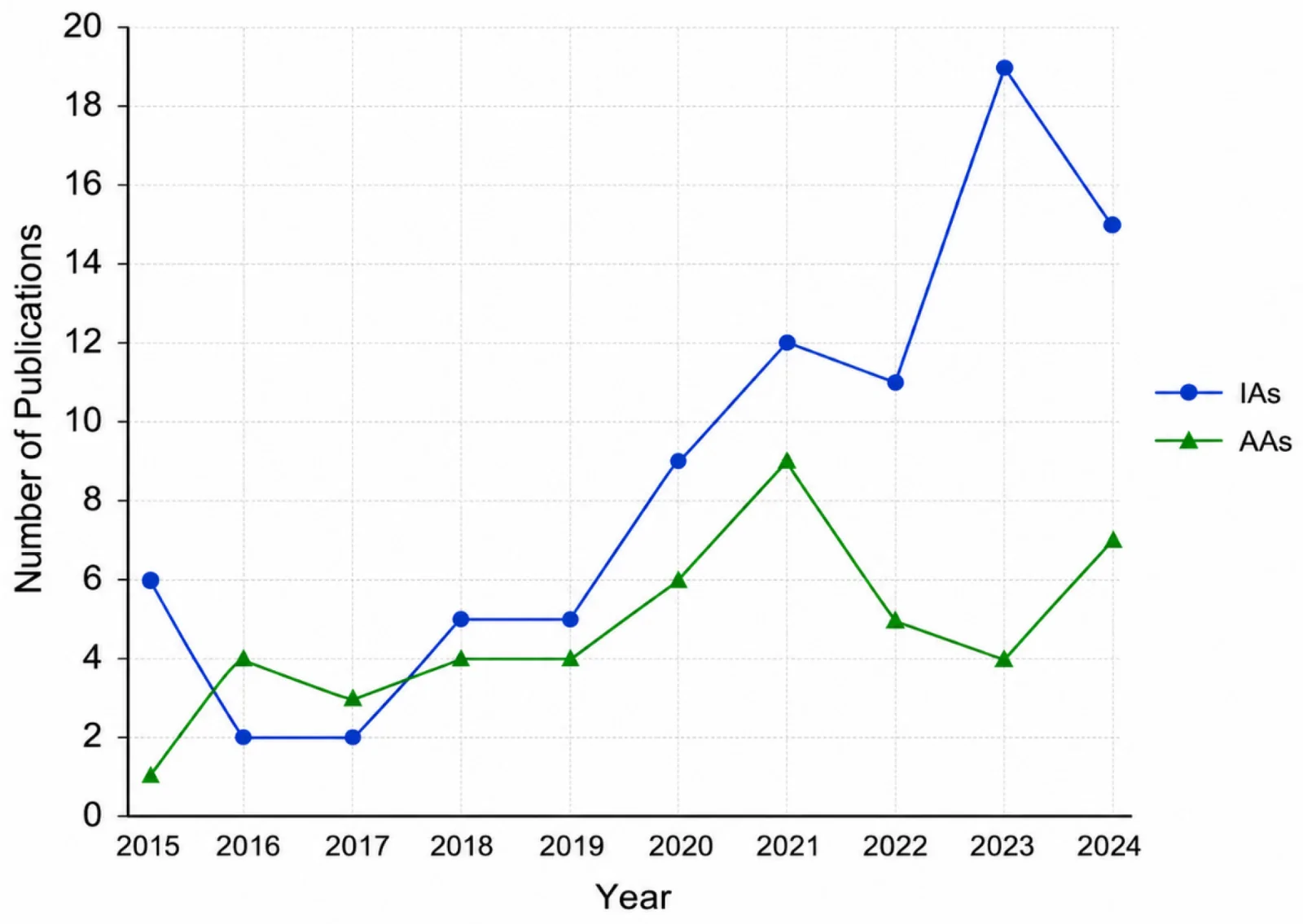
Temporal trends in transcriptomics-related publications on IAs and AAs. The number of scientific publications reporting transcriptomic data associated with IA (blue) and AA (green) biosynthesis is shown for the period 2015–2024. Data were obtained from a systematic search in the Web of Science database. Both alkaloid classes show a general increase in transcriptomics-related publications over time, reflecting the expanding use of omics approaches in plant specialized metabolism. The higher number of IA-related studies compared with AA-related studies indicates an imbalance in research activity within the scope of this review but should not be interpreted as a direct measure of biological importance or overall relevance. Despite the increase in transcriptomic datasets, functional characterization of biosynthetic genes remains limited, indicating that data generation continues to outpace mechanistic validation.

Isoquinoline alkaloids (IAs) are widely distributed across multiple plant families and exhibit remarkable structural diversity, which has driven extensive research into their biosynthesis. Transcriptomic studies have been reported for more than 70 species across approximately 20 plant families, reflecting the growing use of omics approaches to investigate the genetic basis of IA production. A systematic search of the Web of Science database identified 237 publications related to IA transcriptomics, of which 104 focused specifically on plant systems (Figure 5). Over the last decade, the number of studies has generally increased, indicating the expanding application of transcriptomic and multi-omics strategies in alkaloid research.

However, publication volume should be interpreted with caution. The number of studies does not necessarily reflect intrinsic scientific importance, but may also depend on model-species availability, genomic resources, analytical standards, funding priorities, tractable enzyme assays, and the feasibility of heterologous reconstruction. Therefore, the asymmetry between IA and AA research should be interpreted as a combination of research activity, experimental accessibility, resource availability, and historical progress rather than as a direct measure of biological or pharmacological relevance.

To avoid treating transcriptomic studies as a simple inventory, this section organizes IA gene discovery according to the type of evidence generated. Broad transcriptomic and genomic studies have expanded candidate gene repertoires across IA-producing lineages, whereas integrative studies combining transcriptomics with metabolomics, spatial information, inducible systems, or comparative genomics have provided stronger links between gene expression and alkaloid accumulation. Functional studies using heterologous expression and enzymatic assays have converted selected candidates into experimentally validated pathway enzymes. This distinction is important because the number of omics datasets does not necessarily reflect the degree of pathway resolution.

Table 2 summarizes representative studies that illustrate how different omics-guided strategies have contributed to IA pathway elucidation. Rather than comparing sequencing platforms, the table emphasizes the biological question, gene discovery strategy, key findings, and level of functional validation. This organization highlights the gap between large-scale candidate identification and experimental confirmation of enzyme function.

The studies summarized in Table 2 show that IA transcriptomic resources have contributed unevenly to pathway elucidation. Broad transcriptome and genome analyses have been valuable for identifying candidate enzyme families such as CYPs, *O*-methyltransferases (OMTs), *N*-methyltransferases (NMTs), reductases, and oxidases, but their mechanistic impact depends on the level of supporting evidence. Studies based mainly on homology searches expand candidate repertoires, whereas studies integrating metabolomics, spatial localization, inducible systems, phylogenetic analysis, and biochemical assays provide stronger pathway assignments [37,41,42,53].

For example, elicitor-induced transcriptome and proteome profiling in *Papaver somniferum* cell cultures linked sanguinarine accumulation with specific transcripts and proteins, illustrating the value of experimentally activating alkaloid metabolism before candidate selection [41]. Similarly, RNA-seq, UPLC-TOF-MS metabolomics, and MALDI-MS imaging in *Podophyllum hexandrum* and *P. peltatum* provided evidence for an overlooked aporphine-related alkaloid pathway and showed how spatial co-localization of transcripts and metabolites can reveal hidden pathway capacity [42]. More recent work in *Coptis chinensis*, *Stephania tetrandra*, *Corydalis yanhusuo*, and *Lophophora williamsii* further illustrates a methodological gradient from candidate prioritization to direct functional validation, including in vitro enzyme assays, gene cloning, heterologous expression, and enzymatic characterization [55,56,57,58].

Following this evidence of hierarchy, RNA-seq-based transcriptomics should be understood as an initial layer of IA gene discovery rather than as a definitive functional assignment tool. Developmental transcriptomic analysis in *Corydalis yanhusuo* revealed dynamic changes in gene expression associated with alkaloid accumulation, supporting transcriptional regulation during organ development [59]. Comparative transcriptomic and metabolomic analyses in *Coptis chinensis* showed that older rhizomes accumulate higher levels of IAs and identified 36 differentially expressed genes associated with the biosynthetic pathway [60]. However, differential expression, co-expression, and metabolite correlation remain forms of candidate prioritization; biochemical function must still be established through cloning, heterologous expression, substrate testing, and enzyme assays.

This section therefore does not aim to provide an exhaustive gene-by-gene inventory of IA biosynthesis, as several reviews have already summarized the stepwise enzymology of well-characterized BIA branches, including morphinan, protoberberine, benzophenanthridine, and aporphine-related pathways [7,37,61,62]. Instead, we focus on how omics-guided strategies have contributed to gene discovery, candidate prioritization, and functional validation across representative IA-producing systems. Representative characterized enzyme modules are summarized in Table 1, and detailed examples with species, substrates, products, discovery methods, and validation strategies are provided in Supplementary Table S1.

Overall, the main bottleneck in IA biosynthesis is not the absence of omics data, but the uneven conversion of candidate genes into experimentally validated biochemical functions. This is particularly relevant for IA scaffold classes that remain less resolved than morphinan, protoberberine, benzophenanthridine, and aporphine-related pathways, where many candidates still require biochemical validation, substrate-specificity testing, and pathway-level reconstruction.

A useful historical reference point for interpreting the IA–AA comparison is provided by Diamond and Desgagné-Penix [62], who framed plant isoquinoline alkaloids (PIAs) as a broad group including benzylisoquinoline, phenethylisoquinoline, ipecac, and Amaryllidaceae alkaloids. At that time, more than 30 biosynthetic genes had been reported for benzylisoquinoline alkaloid pathways, particularly in morphinan, protoberberine, benzophenanthridine, and aporphine-related systems, whereas only five genes had been reported for ipecac alkaloids, one for Amaryllidaceae alkaloids, and none for phenethylisoquinoline alkaloids [62]. This comparison is important because it shows that the apparent maturity of “IA biosynthesis” largely reflects progress in a few models of BIA branches rather than equivalent resolution across all IA-derived alkaloid classes. It also provides a baseline from which recent advances in AA biosynthesis can be evaluated.

### 2.2. Omics-Guided Discovery and Functional Validation of AA Biosynthetic Genes

Amaryllidaceae alkaloid (AA) gene discovery has followed a more targeted and function-driven trajectory than IA gene mining. Early transcriptomic studies in *Lycoris* and *Narcissus* generated candidate repertoires for precursor supply, *O*- and *N*-methylation, CYP-mediated oxidation, and downstream tailoring, but most of these candidates initially lacked direct biochemical validation [63,64,65,66]. Subsequent work progressively transformed these candidate lists into experimentally supported pathway steps through cloning, heterologous expression, purified enzyme assays, isotope-labeled precursor feeding, protein–protein interaction assays, CYP regioselectivity analysis, and transient reconstruction in *Nicotiana benthamiana* [26,27,28,29,30,31,32,33,34].

Early efforts in AA research were driven by transcriptomic analyses aimed at identifying candidate genes involved in alkaloid biosynthesis. The first de novo transcriptome assembly in *Lycoris aurea* provided a comprehensive dataset of candidate genes associated with phenylpropanoid and alkaloid metabolism, including PAL, TYDC, OMTs, CYPs, and NMTs [63]. Subsequent studies improved candidate selection by integrating transcriptomic data with metabolite profiling and differential gene expression analyses across tissues and developmental stages. In *Narcissus pseudonarcissus* ‘King Alfred’, Singh and Desgagné-Penix generated a comprehensive transcriptome and metabolome across different tissues, identifying transcripts corresponding to proposed AA biosynthetic genes such as TYDC, PAL, C4H, C3H, N4OMT, and NR [64]. Similarly, Hotchandani et al. analyzed *Narcissus papyraceus* across developmental stages and showed that AA accumulation and biosynthetic gene expression vary according to tissue and developmental stage, with lycorine as the predominant detected alkaloid in that system [65]. Differential transcriptomics between field-derived bulb tissue and in vitro callus further showed that genes associated with later alkaloid biosynthetic steps, including CYPs and OMTs, were mainly upregulated in field samples, whereas genes related to precursor metabolism were more active in callus [66].

In contrast to IA biosynthesis, where gene discovery is often driven by large-scale omics datasets, AA research has been characterized by a strong emphasis on functional validation. A key milestone was the identification of norbelladine 4′-*O*-methyltransferase (N4OMT) using RNA-seq, co-expression analysis, candidate gene cloning, heterologous expression in *Escherichia coli*, protein purification, and kinetic characterization [30]. This was followed by the discovery of CYP96T1, a cytochrome P450 responsible mainly for the para–para′ oxidative phenol coupling of 4′-*O*-methylnorbelladine, with minor para–ortho′ activity, and by the identification of noroxomaritidine/norcraugsodine reductase (NR), a short-chain dehydrogenase/reductase involved in downstream reduction reactions [28,31].

Recent studies have expanded AA pathway characterization beyond N4OMT, NBS/NR, and CYP96T-mediated oxidative coupling. Mehta et al. [32] identified and functionally tested downstream tailoring enzymes from *Narcissus* cv. Tête-à-Tête, including NtNMT1, a coclaurine-like *N*-methyltransferase associated with galanthamine biosynthesis, together with NtOMT1, NtAKR1, NtSDR1/2, NtCYP71DW1, and NtODD2. In parallel, Karimzadegan et al. [25] characterized phenylpropanoid-pathway CYPs from *Leucojum aestivum*, including LaeC4H/CYP73A and LaeC3′H/CYP98A, supporting the involvement of upstream phenylpropanoid metabolism in the formation of the 3,4-DHBA precursor, although the final enzymatic route to 3,4-DHBA remains unresolved.

The formation of norbelladine has been a central focus because it represents the entry point into AA biosynthesis. Earlier models proposed that tyramine and 3,4-dihydroxybenzaldehyde (3,4-DHBA) condense to form the imine norcraugsodine, which is then reduced to norbelladine. NBS was later identified as an AA-specific enzyme capable of catalyzing this condensation step [26,27]. More recent work showed that NBS and NR do not simply act as isolated enzymes but can function cooperatively. Majhi et al. [29] demonstrated that NBS and NR homologs from *Narcissus papyraceus* and *Leucojum aestivum* physically interact in yeast and in planta, co-localize in the cytoplasm and nucleus, and together increase norbelladine production, supporting the existence of a metabolon-like organization in early AA biosynthesis [29].

Additional studies have expanded the functional characterization of upstream precursor-forming enzymes. Wang et al. [67] cloned and characterized LaTYDC1 from *Lycoris aurea*, demonstrating its role in converting tyrosine into tyramine and showing that its expression is regulated by methyl jasmonate and miR396 [67]. More recently, Karimzadegan et al. characterized phenylpropanoid-pathway enzymes from *Leucojum aestivum*, including cinnamate 4-hydroxylase (CYP73A) and *p*-coumaroyl shikimate/quinate 3′-hydroxylase (CYP98A), contributing to the understanding of 3,4-DHBA precursor formation while leaving the final enzymatic route unresolved [25].

A major methodological advance was provided by Mehta et al. [32], who demonstrated that metabolite accumulation patterns alone can be misleading for gene discovery because AAs may accumulate in tissues where active biosynthesis is no longer occurring. Using [^2^H]-4′-*O*-methylnorbelladine feeding, LC-MS, DESI-MS imaging, and RT-qPCR, they showed that active AA biosynthesis in *Narcissus* cv. Tête-à-Tête is concentrated in young leaf-base tissues, despite broader alkaloid accumulation in mature tissues. This isotope-guided identification of biosynthetically active tissue-enabled RNA-seq and PacBio Iso-Seq-based candidate discovery, leading to the identification of CYP96T homologs involved in alternative oxidative coupling modes and enzymes required for the haemanthamine and galanthamine branches, including NtCYP96T5, NtCYP96T6, NtSDR1/2, NtCYP71DW1, NtODD2, NtOMT1, NtNMT1/coclaurine-like NMT, and NtAKR1 [32].

Complementary evidence from *Lycoris aurea* further supports the central role of CYP96T diversification in AA scaffold formation. Liu et al. [33] generated a PacBio SMRT full-length transcriptome and integrated RNA-seq datasets from different tissues and methyl jasmonate treatments to identify CYP96T1-like candidates. Functional characterization in *Nicotiana benthamiana* showed that LauCYP96T1 and LauCYP96T1-like-2 preferentially catalyze para–para′ oxidative coupling of 4′-*O*-methylnorbelladine, whereas LauCYP96T1-like-1 and LauCYP96T1-like-3 favor para–ortho′ coupling. Through chimera construction, mutagenesis, homology modeling, and docking analyses, this study suggested that specific amino acid residues contribute to regioselectivity differences among closely related CYP96T enzymes [33].

Transient expression systems have now become particularly powerful for testing pathway hypotheses and reconstructing AA biosynthetic branches. *Agrobacterium tumefaciens*-mediated transient expression in *Nicotiana benthamiana* allows multiple candidate enzymes to be tested simultaneously in planta, facilitating the functional assignment of enzymes that act sequentially or cooperatively. In Mehta et al. [32], this strategy enabled the identification and reconstruction of core reactions leading to haemanthamine and galanthamine, including alternative oxidative coupling reactions catalyzed by CYP96T homologs. Similarly, Lamichhane et al. [34] reconstituted key steps of AA biosynthesis in *N. benthamiana* using enzymes from *Leucojum aestivum*. In that study, LaNBS and LaNRII converted tyramine and 3,4-DHBA to norbelladine, LaN4′OMT methylated norbelladine to 4′-*O*-methylnorbelladine, and co-agroinfiltration with LaCYP96T1 or LaCYP96T2 produced phenol-coupled products associated with lycorine-, galanthamine-, and crinine/haemanthamine-type alkaloids [32,34]. Together, these studies represent a major transition from candidate gene discovery toward pathway reconstruction and synthetic biology applications.

The studies summarized in Table 3 illustrate a clear methodological progression in AA research: (i) initial transcriptomic surveys for candidate gene identification [63,64], (ii) integration of metabolomic and expression data to refine candidates [64,65,66,68,69], (iii) functional validation through cloning, heterologous expression, and enzymatic assays [25,27,28,29,30,31,67], (iv) isotope-guided identification of biosynthetically active tissues followed by long-read transcriptomics and candidate prioritization [32], (v) analysis of enzyme cooperation, subcellular organization, and regioselectivity [29,33], and (vi) pathway reconstruction using heterologous plant systems [32,34].

Recent compilations of Amaryllidaceae transcriptomic resources show that the number of available datasets is larger than previously recognized, spanning genera such as *Crinum*, *Galanthus*, *Hippeastrum*, *Leucojum*, *Lycoris*, *Narcissus*, and *Zephyranthes*. However, these resources are unevenly distributed across the family and should not be interpreted as equivalent to functional pathway elucidation. Therefore, Table 3 distinguishes transcriptomic resources used for candidate discovery from studies that provide biochemical validation or pathway reconstruction.

Rather than adding another chronological inventory, Table 3 should be interpreted as a comparison of evidence levels in AA pathway elucidation. Early transcriptomic studies mainly generated candidate repertoires, whereas later studies progressively increased the strength of functional evidence by incorporating recombinant enzyme assays, isotope-labeled precursor feeding, protein–protein interaction assays, CYP regioselectivity tests, and transient pathway reconstruction in *Nicotiana benthamiana*. This progression shows that the major advance in AA research has not been the accumulation of transcriptomes alone, but the transition from candidate identification to experimentally testable pathway modules. Nevertheless, Table 3 also highlights persistent gaps, particularly the unresolved enzymatic formation of 3,4-DHBA, incomplete characterization of downstream tailoring reactions, and limited functional validation across the broader diversity of Amaryllidoideae.

## 3. Integrative Approaches to Biosynthetic Understanding

The comparison between IAs and AAs provides an interpretive framework for understanding how plant lineages transform aromatic amino acid-derived precursors into structurally complex alkaloids. Rather than focusing only on whether individual genes have been identified, an integrative view considers precursor origin, scaffold-forming chemistry, enzyme recruitment, substrate flexibility, tissue specificity, and pathway reconstruction together.

A key mechanistic contrast lies in the first scaffold-forming step. In BIA biosynthesis, dopamine and 4-hydroxyphenylacetaldehyde (4-HPAA), both derived from L-tyrosine metabolism, are condensed by norcoclaurine synthase (NCS) through a stereoselective Pictet–Spengler reaction to produce (*S*)-norcoclaurine, the central entry intermediate of BIA biosynthesis [13,37]. In AA biosynthesis, tyramine and 3,4-dihydroxybenzaldehyde (3,4-DHBA) are instead joined through NBS-mediated imine formation to generate norcraugsodine, followed by NR-mediated reduction to norbelladine [26,27,29]. This difference reflects the chemistry of the amine precursors: dopamine contains a catechol ring that can support Pictet–Spengler cyclization, whereas tyramine lacks the additional ortho-hydroxyl group and therefore proceeds through a linear norbelladine intermediate rather than immediate ring closure.

A second integrative axis is oxidative phenol coupling. In IAs, CYP80 and CYP719 enzymes participate in branch-specific oxidative transformations, including aporphine formation, salutaridine formation, and methylenedioxy bridge formation [36,37]. In AAs, CYP96T homologs catalyze regioselective oxidative coupling of 4′-*O*-methylnorbelladine (4′-OMN), directing metabolism toward haemanthamine/crinine-, galanthamine-, or lycorine-type scaffolds [31,32,33,34]. This comparison shows that similar catalytic logic—oxidative C–C bond formation—can be implemented by different CYP families acting on different substrates. Therefore, pathway similarity should be interpreted at the level of catalytic strategy rather than direct enzyme equivalence.

One major unresolved gap in AA biosynthesis remains in the enzymatic formation of 3,4-DHBA. Current evidence supports the involvement of upstream phenylpropanoid metabolism, including C4H/CYP73A and C3′H/CYP98A activities, but the direct enzymatic steps leading to 3,4-DHBA remain unknown [24,25,70]. Proposed routes may involve caffeic acid- or caffeoyl-shikimate-related intermediates, or an alternative route through 4-hydroxybenzaldehyde. Therefore, 3,4-DHBA formation should be presented as an evidence-weighted hypothesis rather than a fully established pathway.

Recent progress in CYP96T characterization also reinforces the importance of looking beyond simple homology-based annotation. Closely related CYP96T homologs can catalyze different regioselective couplings, showing that small changes in substrate positioning or active-site architecture can redirect scaffold formation [32,33]. Similar caution applies to less-resolved IA branches, where functionally analogous oxidative transformations may remain unassigned. Thus, future candidate searches should combine chemical logic, metabolite structure, phylogenetic relationships, tissue-specific expression, and functional assays rather than relying on homology alone [32,33,37,42].

Enzyme promiscuity and substrate flexibility should also be considered when interpreting pathway evolution. Multiple-substrate testing has already been reported for enzymes in both IA and AA biosynthesis, particularly among CYPs, methyltransferases, and reductases. In AAs, CYP96T1 and related CYP96T homologs have been tested with multiple AA-related substrates and can generate alternative phenol-coupled products, while NR-related enzymes can reduce more than one imine or alkaloid substrate [28,29,31,33,34]. In IAs, substrate flexibility has also been reported for methyltransferases and CYP-mediated tailoring enzymes, contributing to scaffold diversification [37,57]. Thus, the remaining limitation is not the absence of promiscuity testing, but the lack of systematic cross-pathway substrate panels comparing structurally related IA and AA intermediates.

Together, these examples suggest that alkaloid pathway evolution may arise less from the invention of entirely new catalytic chemistries than from the repeated recruitment, duplication, and functional divergence of a limited set of catalytic modules, including amine–aldehyde condensation enzymes, *O*- and *N*-methyltransferases, reductases, CYPs, and other oxidative enzymes. In IAs, broad taxonomic distribution has enabled diversification into multiple scaffold classes, including morphinan, protoberberine, benzophenanthridine, aporphine, phthalideisoquinoline, ipecac, and bisbenzylisoquinoline-type alkaloids. In AAs, a more lineage-restricted system has generated scaffold diversity mainly through diversification of 4′-OMN coupling and downstream tailoring reactions. This modular perspective helps explain how changes in substrate availability, enzyme regioselectivity, and branch-point control can produce large structural outcomes from relatively conserved biochemical operations [11,32,33,34,37].

The different taxonomic distributions of IAs and AAs also have methodological implications for pathway discovery. IAs are broadly distributed across multiple plant families, especially within Ranunculales, which has facilitated comparative genomics, cross-species transcriptome mining, and recognition of conserved enzyme families across related but chemically diverse taxa [9,37,53,71]. By contrast, AAs are largely restricted to Amaryllidaceae/Amaryllidoideae, and functional studies have concentrated mainly on a few genera, including *Narcissus*, *Lycoris*, and *Leucojum* [27,32,64,65]. This narrower taxonomic and genomic sampling may partly explain why AA biosynthesis remained chemically characterized but molecularly unresolved for longer. At the same time, the discovery of functionally divergent CYP96T homologs shows that restricted taxonomic distribution does not imply limited biochemical innovation; rather, AAs illustrate how a lineage-restricted pathway can generate substantial scaffold diversity through diversification of a relatively small set of pathway-specific enzymes [32,33,34].

**Figure 6:**
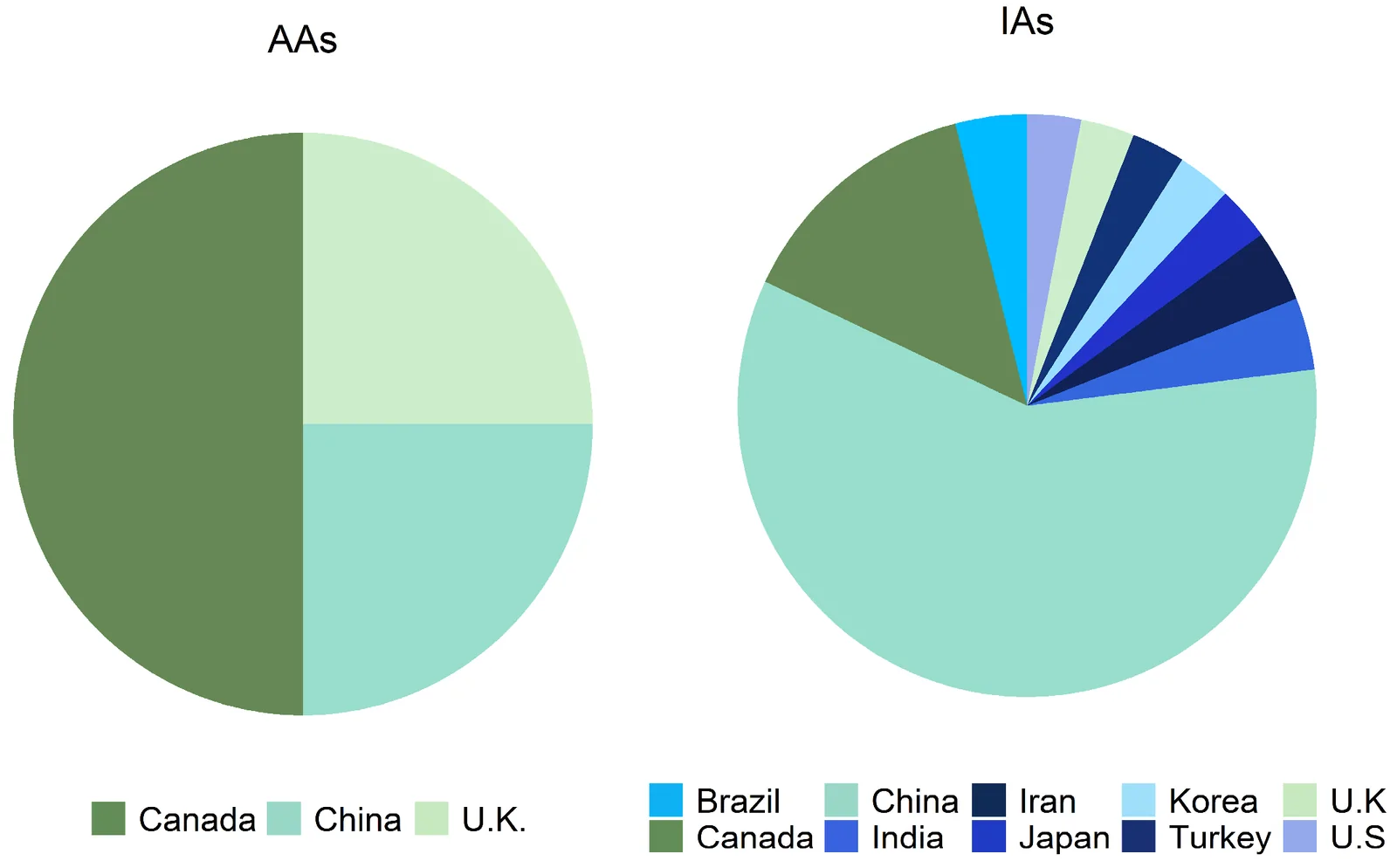
Geographic distribution of sequencing studies on IA- and AA-producing plants. The figure summarizes the countries associated with transcriptomic or genomic sequencing studies of plants producing IAs and AAs. IA-related studies show a broader international distribution, whereas AA-related sequencing efforts are currently concentrated in fewer countries, mainly Canada, China, and the United Kingdom. This geographic imbalance reflects the more restricted taxonomic and institutional scope of AA omics research within the dataset analyzed here and highlights the need for broader global sampling to improve pathway discovery and comparative biosynthetic analyses.

These taxonomic differences are also reflected in the geographic distribution of sequencing efforts. Studies on IA-producing plants have been conducted across a broader set of countries, whereas AA transcriptomic studies remain concentrated in fewer regions, mainly Canada, China, and the United Kingdom (Figure 6). This imbalance is important because limited geographic and taxonomic sampling may constrain the discovery of pathway variants, lineage-specific enzymes, and novel alkaloid chemotypes. Expanding research to additional Amaryllidaceae-rich regions and species would strengthen comparative analyses and improve the discovery of enzymes with biotechnological potential [27,33,64,65].

Beyond enzyme discovery and pathway reconstruction, regulatory mechanisms are increasingly recognized as important determinants of alkaloid biosynthesis. In IA-producing species, alkaloid accumulation is influenced by elicitor-responsive transcriptional programs, tissue specificity, developmental context, and genome-level changes. In *Papaver somniferum*, genome-scale analyses have shown that whole-genome duplication, genome rearrangements, and gene fusion contributed to the evolution of specialized BIA metabolism, including the organization of genes associated with morphinan and related branches [53,71]. Transcriptomic studies in IA-producing plants have also identified transcription factor families such as WRKY, MYB, bHLH, and AP2/ERF as candidates associated with alkaloid biosynthesis and elicitor responses, although many of these regulatory relationships remain inferred from expression patterns rather than functionally validated.

In AAs, regulatory knowledge remains more limited, but primary studies have begun to identify specific control points. In *Lycoris aurea*, methyl jasmonate (MeJA) treatment induced broad transcriptional reprogramming, including differentially expressed genes related to secondary metabolism, transcription factors, and transporters [72]. Many WRKY, MYB, and bHLH/MYC candidates were upregulated under MeJA treatment, suggesting that jasmonate-responsive transcriptional regulation contributes to AA pathway control. A later functional study identified LaTYDC1, a tyrosine decarboxylase involved in tyramine formation for galanthamine biosynthesis, and showed that both LaTYDC1 expression and tyramine content increased under MeJA treatment. The same study demonstrated that miR396 targets LaTYDC1 and promotes degradation of its mRNA, revealing a post-transcriptional regulatory layer affecting precursor supply in AA biosynthesis [67].

More recently, Zhou et al. [73] provided the strongest functional evidence to date for transcriptional regulation of AA biosynthesis. Using comparative transcriptomics of MeJA-treated *L. aurea* seedlings, they identified 31,193 differentially expressed genes (DEGs) and 732 differentially expressed transcription factors (TFs) belonging to 51 TF families; among these, 41 TF candidates were correlated with lycorine accumulation. The most abundant MeJA-responsive TF families included WRKY, AP2/ERF-ERF, MYB, bHLH, and NAC. Zhou et al. then cloned and characterized LaMYC2, a MeJA-inducible bHLH/MYC transcription factor localized in the nucleus. LaMYC2 interacted with LaJAZ proteins, transient overexpression of LaMYC2 in *L. aurea* petals increased lycorine content, and LaMYC2 overexpression activated LaPAL and LaTYDC transcript levels. Furthermore, yeast one-hybrid assays showed that LaMYC2 binds the E-box motif in the LaTYDC promoter, supporting its role as a positive regulator of lycorine biosynthesis [73].

**Figure 7:**
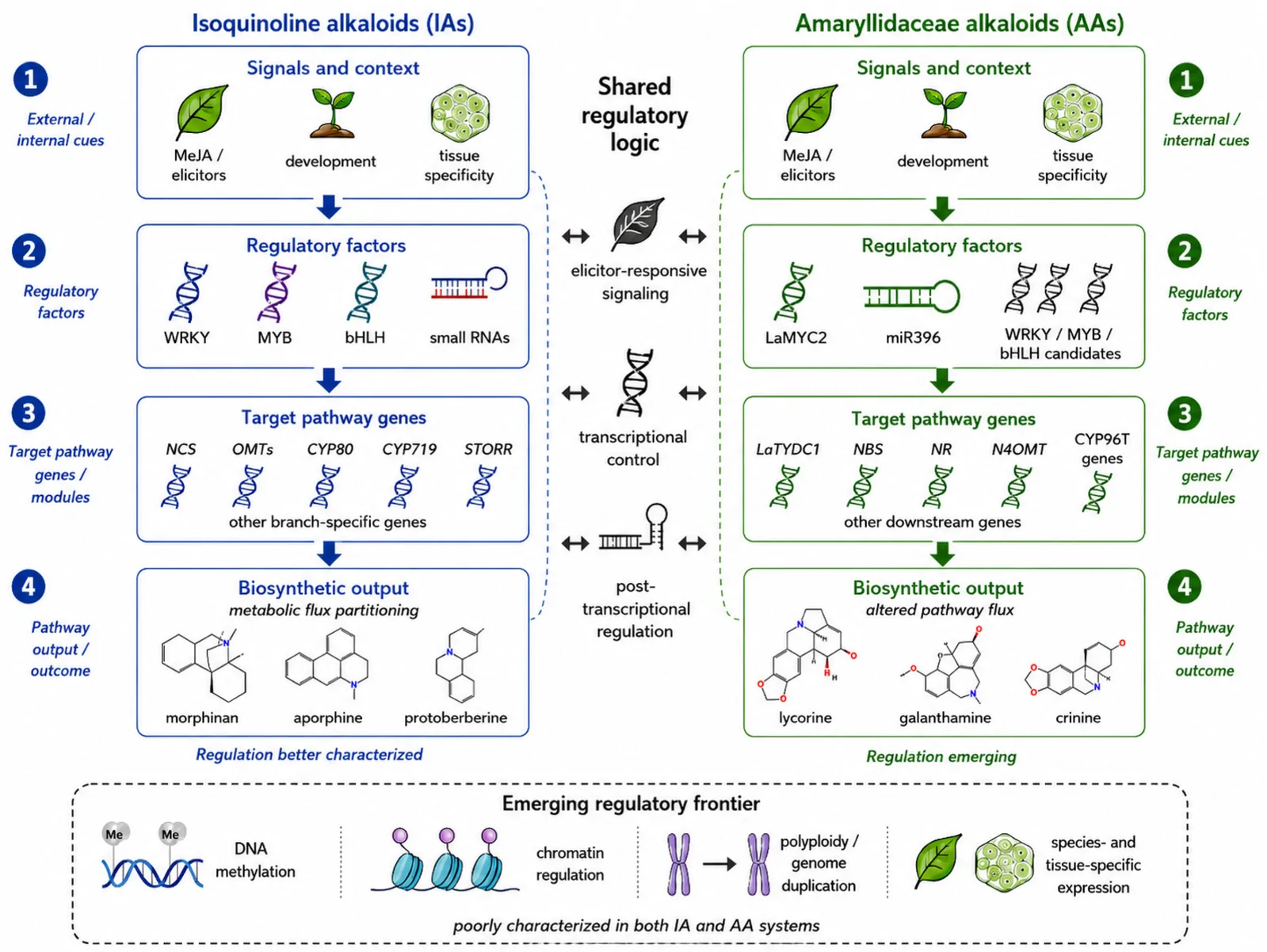
Regulatory layers influencing IA and AA biosynthesis. The figure summarizes regulatory levels currently associated with IA and AA biosynthesis, including elicitor-responsive signaling, transcriptional regulation, post-transcriptional control, and emerging epigenetic influences. In IA-producing species, jasmonate-responsive transcription factor families such as WRKY, MYB, bHLH, and AP2/ERF have been associated with modulation of structural biosynthetic genes, although many relationships remain inferred from expression data. In AA-producing species, methyl jasmonate-responsive transcriptomic studies in *Lycoris aurea* have identified WRKY, AP2/ERF, MYB, bHLH, and NAC candidates associated with lycorine accumulation. Functional evidence supports regulation of LaTYDC1 by MeJA and miR396, as well as the role of LaMYC2 as a positive regulator of lycorine biosynthesis through interaction with LaJAZ proteins and activation of LaPAL and LaTYDC expression. Epigenetic regulation is presented as an emerging but still poorly characterized layer in both alkaloid groups.

Epigenetic regulation remains far less explored in both IA and AA biosynthesis and should therefore be presented as an emerging frontier rather than an established regulatory mechanism. Nevertheless, this layer is biologically relevant because genome duplication, polyploidy, chromatin accessibility, and DNA methylation can influence gene dosage, paralog expression, and tissue-specific transcriptional programs. This may be particularly important in alkaloid-producing lineages with complex genomes. For example, genome analyses in *Papaver somniferum* have shown that whole-genome duplication, genome rearrangements, and gene fusion contributed to the evolution of BIA metabolism [53,71], while phylogenomic analyses in Amaryllidaceae have highlighted polyploidy as an important evolutionary force in the diversification of some Amaryllidoideae lineages [74]. However, direct evidence linking DNA methylation or chromatin remodeling to IA or AA pathway regulation remains limited. Thus, epigenetic regulation should be considered a promising but still poorly characterized layer for future investigation. These regulatory layers are summarized in Figure 7.

Overall, integrative approaches that combine transcriptomics, metabolomics, biochemical validation, isotope tracing, tissue-specific analyses, regulatory studies, and comparative chemical reasoning are essential for moving beyond candidate gene lists toward mechanistic pathway understanding. The comparison between IAs and AAs shows that the central challenge is no longer only to generate sequencing data, but to connect omics information with enzyme function, pathway architecture, metabolite flux, tissue specificity, regulation, and evolutionary diversification [32,33,34,37,57,58].

From this perspective, IAs and AAs illustrate complementary discovery challenges. IAs, especially BIAs, benefit from broader taxonomic distribution and comparative omics across families such as Papaveraceae, Ranunculaceae, Berberidaceae, and Menispermaceae, but many non-model scaffold classes still require functional validation and pathway-level reconstruction. AAs, in contrast, are concentrated mainly within Amaryllidaceae/Amaryllidoideae, where recent work has shown that lineage-restricted pathways can generate substantial chemical diversity through functional divergence of CYP96T homologs, NBS/NR cooperation, downstream tailoring reactions, and active-tissue-specific biosynthesis [32,33,34,64]. Thus, future progress in both alkaloid groups will depend on combining broad comparative sampling with experimentally testable biochemical hypotheses.

## 4. Challenges and Opportunities

The increasing availability of sequencing technologies has greatly accelerated the study of alkaloid biosynthesis. DNA sequencing, long-read transcriptomics, RNA-seq, metabolomics, and functional assays now provide complementary layers of information for identifying and validating biosynthetic genes. However, the transition from candidate gene discovery to complete pathway reconstruction remains challenging, particularly in plant specialized metabolism, where gene families are often large, substrates are structurally similar, and biosynthetic activity may be restricted to specific tissues or developmental stages.

One of the main technical challenges is the complexity of plant genomes. Many alkaloid-producing plants have large genomes, high transposable element content, heterozygosity, and frequent polyploidy, all of which complicate genome assembly and gene annotation. Polyploid genomes are especially difficult to resolve because highly similar paralogous or homologous sequences may lead to fragmented assemblies, chimeric contigs, switch errors, or inaccurate gene models. This issue is particularly relevant for several IA- and AA-producing lineages, including Ranunculaceae and Amaryllidaceae, where genome duplication and polyploidy have contributed to species diversification and may also influence biosynthetic gene-family expansion and metabolic diversity. In this context, long-read sequencing, phased genome assembly, and full-length transcriptomics represent important opportunities for improving the identification of pathway genes and distinguishing closely related paralogs or homeologs.

These challenges are directly connected to alkaloid biosynthesis. In IAs, genomic and transcriptomic resources have enabled broad comparative analyses across multiple plant families and have contributed to the identification of enzyme families involved in key transformations, including methyltransferases, cytochrome P450s, reductases, dioxygenases, and other tailoring enzymes. However, these enzymes have not been studied uniformly across plant lineages. For example, morphinan-related enzymes such as STORR, T6ODM, CODM, COR, and neopinone isomerase have been primarily characterized in Papaveraceae, especially *Papaver somniferum*, whereas protoberberine- and aporphine-related enzymes such as CYP80G2, CYP719 enzymes, and selected OMTs have been investigated mainly in Berberidaceae and Ranunculaceae species, including *Coptis japonica* and *Coptis chinensis*. In *Papaver somniferum*, genome-scale analyses have also shown that gene duplication and genome rearrangements are associated with the evolution and diversification of BIA biosynthesis [53,72]. In contrast, AA-producing plants have fewer genomic resources, and most functional advances have been achieved through targeted transcriptomics, metabolomics, and biochemical validation in a limited number of genera, mainly *Narcissus*, *Lycoris*, and *Leucojum*.

Despite this narrower taxonomic scope, AA research has recently advanced rapidly. The functional characterization of core AA enzymes, including N4OMT, CYP96T1, NR, and NBS, transformed the field from candidate gene discovery toward mechanistic and pathway-level understanding [26,27,28,30,31]. More recent studies have expanded this framework by showing that NBS and NR may act cooperatively in early norbelladine formation, that CYP96T homologs can control regioselective oxidative coupling, and that active biosynthesis can be spatially resolved using isotope-labeled precursors and tissue-specific transcriptomics [29,32,33]. These findings demonstrate that the main limitation is no longer the absence of transcriptomic datasets alone, but rather the need to connect gene expression, metabolite flux, enzyme activity, localization, and pathway architecture.

An important unresolved challenge in AA biosynthesis remains the origin of 3,4-dihydroxybenzaldehyde (3,4-DHBA), the aldehyde precursor that condenses with tyramine to form norbelladine. Although phenylpropanoid intermediates have long been proposed as possible precursors, the complete enzymatic sequence leading from L-phenylalanine-derived metabolites to 3,4-DHBA has not yet been established. Recent work in *Leucojum aestivum* clarified part of the upstream phenylpropanoid route by functionally characterizing LaeC4H/CYP73A and LaeC3′H/CYP98A phenylpropanoid-pathway CYPs; however, these enzymes explain only early hydroxylation steps and do not account for the formation of 3,4-DHBA itself [25]. LaeC3′H/CYP98A hydroxylates *p*-coumaroyl shikimate rather than free *p*-coumaric acid or 4-hydroxybenzaldehyde, indicating that direct conversion of these proposed intermediates to 3,4-DHBA remains unsupported. Therefore, the phenylpropanoid origin of 3,4-DHBA should be regarded as the most plausible working hypothesis, but not as a fully resolved biosynthetic route (Table 4).

Based on the currently available evidence, 3,4-DHBA formation in Amaryllidaceae should be presented as an unresolved, evidence-weighted hypothesis rather than as a fully established pathway. Current data support the involvement of upstream phenylpropanoid metabolism, including C4H/CYP73A and C3′H/CYP98A activities, but the enzymatic steps directly leading to 3,4-DHBA remain unknown. Proposed routes may involve *p*-coumaroyl- or caffeoyl-shikimate-like intermediates, caffeic acid-related intermediates, or an alternative route through 4-hydroxybenzaldehyde; however, none of these routes have been completely demonstrated.

The missing steps are likely to require one or more still-unidentified enzymes capable of side-chain shortening, oxidation, dehydrogenation, lyase activity, peroxidase-like reactions, or hydroxybenzaldehyde synthase-like activity. Candidate genes involved in 3,4-DHBA formation should therefore be expected to co-express with early AA biosynthetic genes, be enriched in biosynthetically active tissues, respond to developmental or elicitor cues, and correlate with 3,4-DHBA, norcraugsodine, norbelladine, or downstream AA accumulation. Testing this hypothesis will require isotope-labeled precursor feeding, tissue-specific metabolomics, co-expression analysis, and biochemical validation of candidate enzymes. Together, these observations clarify that the main unresolved point is not whether aromatic amino acid-derived precursors feed AA biosynthesis, but which specific enzymatic sequence converts phenylpropanoid-derived intermediates into 3,4-DHBA.

Another major challenge is the functional annotation of large enzyme families. Homology-based annotation is useful for identifying candidate genes, but it is often insufficient to assign biochemical functions, especially cytochrome P450s, *O*- and *N*-methyltransferases, reductases, and dioxygenases. Closely related enzymes may catalyze different reactions, act on different substrates, or display different regioselectivities. This has become particularly clear in AAs, where CYP96T homologs can redirect 4′-*O*-methylnorbelladine toward distinct scaffold types, including haemanthamine-, galanthamine-, and lycorine-related branches [32,33]. Therefore, future studies should combine transcriptomics with substrate-guided enzymology, mutagenesis, protein modeling, metabolomics, and in planta validation to move beyond annotation toward mechanistic understanding.

These challenges also create important opportunities for biotechnology. Fully or partially elucidated alkaloid pathways can be transferred into heterologous systems, including microbial cell factories and transient plant-expression platforms. In IAs, engineered microorganisms have already been used to reconstruct complex branches of benzylisoquinoline alkaloid biosynthesis, demonstrating the feasibility of producing high-value compounds through synthetic biology. By contrast, AA biotechnology remains at an earlier stage. Transient expression in *Nicotiana benthamiana* has recently emerged as a powerful platform for testing candidate genes and reconstructing AA biosynthetic branches, including steps toward galanthamine-, haemanthamine-, and lycorine-type alkaloids, but stable and scalable microbial production of AAs remains less developed [32,34].

This difference reflects the more complete pathway knowledge available for several IA branches, the longer history of enzyme characterization in BIA systems, and unresolved AA-specific bottlenecks, particularly the complete enzymatic route to 3,4-DHBA, early enzyme cooperation in norbelladine formation, and downstream tailoring reactions. A qualitative comparison of the main production platforms for IA and AA compounds is summarized in Table 5.

Chemical synthesis remains an important alternative for producing natural product-derived molecules and analogs, particularly when plant extraction is inefficient or when structural diversification is required. However, structurally complex alkaloids, total or semi-synthetic routes may involve multiple steps, protecting-group strategies, low overall yields, expensive catalysts or reagents, and considerable solvent and waste generation. Therefore, microbial, plant-cell, and transient plant-expression platforms are increasingly explored as potentially more sustainable approaches for producing complex alkaloids and pathway intermediates, especially when biosynthetic enzymes can provide regio- and stereoselective transformations that are difficult to achieve chemically.

Overall, no single production strategy is universally optimal. Plant extraction remains relevant for naturally abundant compounds. In vitro cultures are useful for controlled biosynthetic studies and elicitation. Microbial systems offer the greatest scalability once pathways are sufficiently resolved. Transient plant expression provides a rapid platform for testing complex plant enzymes before transfer into more stable production chassis. Studies comparing field-grown tissues, callus, and developmental stages further show that alkaloid accumulation does not always reflect the site of active biosynthesis, reinforcing the need to integrate metabolomics with isotope tracing and tissue-specific expression analyses, particularly in AAs where young basal tissues may be biosynthetically active even when mature tissues accumulate higher alkaloid levels [32,66].

The main opportunity for the field lies in integrating genomic, transcriptomic, metabolomic, biochemical, regulatory, and synthetic biology approaches into coherent discovery pipelines. For IAs, the priority is to expand functional validation beyond candidate gene lists and resolve remaining pathway gaps across diverse alkaloid branches. For AAs, the priority is to broaden taxonomic and genomic sampling while continuing the function-driven approach that has recently produced major advances. In both cases, future progress will depend on moving from descriptive omics datasets toward experimentally validated, reconstructable, and engineerable biosynthetic pathways. Expanding regulatory studies to include transcription factors, small RNAs, chromatin-level regulation, and elicitor-responsive signaling will further strengthen these pipelines.

## 5. Conclusions

The elucidation of IA and AA biosynthetic pathways illustrates how the study of plant specialized metabolism has shifted from compound isolation, radiotracer experiments, and biochemical inference toward omics-guided discovery, functional validation, and pathway reconstruction. Although both alkaloid groups are connected to aromatic amino acid metabolism and share broad biochemical themes, including amine–aldehyde chemistry, methylation, reduction, hydroxylation, and oxidative coupling, they represent distinct biosynthetic solutions for generating nitrogen-containing scaffolds. In IAs, NCS-mediated Pictet–Spengler cyclization rapidly produces cyclic tetrahydroisoquinoline scaffolds, whereas in AAs, NBS/NR-mediated imine formation and reduction first generate the more flexible intermediate norbelladine, which is subsequently converted into diverse AA scaffolds through oxidative coupling and downstream tailoring.

The comparison between IAs and AAs highlights the importance of enzyme recruitment, functional divergence, and taxonomic context in alkaloid diversification. IA biosynthesis has benefited from broader taxonomic sampling, more extensive genomic and transcriptomic resources, and earlier functional characterization of several major branches. In contrast, AA biosynthesis has advanced more recently through function-driven approaches, isotope-guided identification of biosynthetically active tissues, protein–protein interaction studies, CYP96T regioselectivity analyses, and transient reconstruction in *Nicotiana benthamiana*. These advances show that restricted taxonomic distribution does not imply limited biochemical innovation; rather, Amaryllidaceae species generate structural diversity through lineage-specific diversification of key pathway enzymes and downstream tailoring reactions.

Despite these advances, important gaps remain in both systems. In IA biosynthesis, large omics datasets have generated extensive candidate gene lists, but functional validation is still incomplete for several less-studied scaffold classes. In AA biosynthesis, key unresolved steps include the complete enzymatic route to 3,4-DHBA, the regulatory architecture controlling pathway flux, and the full characterization of downstream tailoring reactions. Recent studies on CYP96T diversification, NBS/NR cooperation, LaMYC2-mediated regulation, and heterologous pathway reconstruction show that the field is moving beyond descriptive transcriptomics toward mechanistic and experimentally testable models. Future progress will depend on integrating comparative omics, isotope-labeled precursor feeding, tissue-specific metabolomics, enzyme assays, regulatory studies, and reconstruction platforms to develop predictive and sustainable strategies for alkaloid biosynthesis.

## Figures and Tables

**Table 1:** Functional comparison of IA and AA biosynthesis, including precursor origin, scaffold-forming chemistry, characterized enzyme modules, and current pathway gaps.

Aspect	Isoquinoline Alkaloids (IAs)	Amaryllidaceae Alkaloids (AAs)
Main taxonomic distribution	Broadly distributed across multiple plant families, including Papaveraceae, Ranunculaceae, Berberidaceae, Menispermaceae, Lauraceae, Annonaceae, and others. Several well-studied BIA-producing families belong to Ranunculales.	Mainly associated with Amaryllidoideae within Amaryllidaceae, with occasional reports in other Asparagales lineages.
Upstream metabolic origin	Derived primarily from shikimate pathway-derived aromatic amino acid metabolism. In canonical BIA biosynthesis, L-tyrosine-derived intermediates provide both the dopamine unit and the aldehyde precursor, such as 4-hydroxyphenylacetaldehyde (4-HPAA).	Derived from two aromatic amino acid-derived routes. L-tyrosine provides tyramine, whereas L-phenylalanine is proposed to contribute to 3,4-dihydroxybenzaldehyde (3,4-DHBA) through the phenylpropanoid pathway. The complete enzymatic route to 3,4-DHBA remains unresolved.
First scaffold-forming reaction	NCS-mediated Pictet–Spengler condensation of dopamine and 4-HPAA to form (*S*)-norcoclaurine.	NBS-mediated imine formation from tyramine and 3,4-DHBA to form norcraugsodine, followed by NR-mediated reduction to norbelladine.
First central intermediate	(*S*)-Norcoclaurine.	Norbelladine.
Main branch-point intermediate	(*S*)-Reticuline, which gives rise to multiple IA branches. In morphinan biosynthesis, STORR converts (*S*)-reticuline to (*R*)-reticuline.	4′-*O*-methylnorbelladine (4′-OMN), formed by N4OMT-mediated 4′-*O*-methylation of norbelladine and preceding regioselective oxidative phenol coupling.
Main scaffold-diversification chemistry	*O*- and *N*-methylation, P450-dependent hydroxylation, oxidative phenol coupling, methylenedioxy bridge formation, oxidation/reduction, dioxygenase-mediated reactions, acetylation, demethylation, and other branch-specific tailoring reactions.	4′-*O*-methylation, CYP96T-mediated regioselective oxidative phenol coupling, reductions, oxidations, additional *O*- and *N*-methylation reactions, methylenedioxy bridge formation, hydroxylations, and other downstream tailoring reactions.
Comparable characterized genes/enzyme modules *	Precursor and entry steps: NCS. Methylation: 6OMT, CNMT, 4′OMT, TNMT and branch-specific OMTs/NMTs. Hydroxylation and oxidative scaffold formation: CYP80B, CYP80G, CYP719, SalSyn/CYP719B1. Branch formation and tailoring: BBE/BBE-like enzymes, CFS, SPS, STORR, SalR, SalAT, T6ODM, CODM, COR, neopinone isomerase/NISO, MSH, P6H, DBOX and other branch-specific reductases, dioxygenases, demethylases, acetyltransferases and oxidases.	Precursor and entry steps: NBS, NR, TYDC, CYP73A/C4H, CYP98A/C3′H and proposed phenylpropanoid-route enzymes. Methylation: N4OMT, NtOMT1, NtNMT1/coclaurine-like NMT and other branch-specific OMTs/NMTs. Oxidative scaffold formation: CYP96T1, CYP96T5, CYP96T6, LauCYP96T1/LauCYP96T1-like homologs, LaCYP96T1/LaCYP96T2 and other characterized CYP96T homologs. Branch formation and tailoring: NtSDR1, NtSDR2, NR/SDR enzymes, NtAKR1, NtCYP71DW1, NtODD2 and additional downstream reductases, hydroxylases, oxidases and tailoring enzymes.
Representative CYP-catalyzed scaffold-forming reactions	CYP80G2 catalyzes intramolecular C–C phenol coupling of (*S*)-reticuline toward aporphine-type alkaloids. CYP719 enzymes participate in branch-specific oxidative transformations, including salutaridine formation and methylenedioxy bridge formation.	CYP96T homologs catalyze regioselective oxidative coupling of 4′-OMN, including para–para′, para–ortho′, and ortho–para′ coupling modes associated with haemanthamine/crinine-, galanthamine-, and lycorine-type scaffolds.
Representative scaffold outcomes	Morphinan-, protoberberine-, tetrahydroprotoberberine-, protopine-, benzophenanthridine-, aporphine-, phthalideisoquinoline-, ipecac-, bisbenzylisoquinoline-, narciclasine-, and related IA scaffolds.	Haemanthamine/crinine-, galanthamine-, lycorine-, tazettine-, montanine-, cherylline-, norbelladine-, and related AA scaffolds.
Current level of pathway elucidation	Several BIA branches, particularly morphinan, protoberberine, benzophenanthridine, and aporphine-related pathways, are relatively well characterized. However, pathway resolution remains uneven across IA scaffold classes and plant lineages.	Rapidly advancing but still incomplete. Recent studies have clarified NBS/NR-mediated norbelladine formation, CYP96T regioselectivity, and several downstream branch-specific steps, but 3,4-DHBA formation and many tailoring reactions remain unresolved.
Dominant discovery approaches	Enzyme purification, radiolabeling, transcriptomics, comparative genomics, homology-based mining, metabolomics, co-expression analysis, mutant analysis, heterologous expression, yeast reconstruction, and biochemical validation.	Transcriptomics, metabolomics, isotope-guided active-tissue selection, co-expression analysis, biochemical validation, protein–protein interaction assays, CYP regioselectivity analysis, transient expression in *Nicotiana benthamiana*, and pathway reconstruction.
Recent pathway refinements	STORR resolved the (*S*)- to (*R*)-reticuline gateway into morphinan biosynthesis; neopinone isomerase clarified late morphinan biosynthesis; engineered yeast systems enabled reconstruction of morphinan and other BIA modules.	NBS/NR cooperation clarified norbelladine formation; multiple CYP96T homologs showed species-dependent regioselectivity; recent studies identified reductases, methyltransferases, AKRs, CYP71DW enzymes, ODDs, and transient reconstruction strategies for galanthamine-, haemanthamine-, and lycorine-related branches.
Current limitations	Broad structural and taxonomic diversity, but uneven pathway resolution across scaffold classes. Many branches remain less characterized than morphinan, protoberberine, benzophenanthridine, and aporphine-related pathways, and functional validation of candidate enzymes remains incomplete in several lineages.	Substantial structural diversity within a more lineage-restricted group, but fewer functionally validated enzymes and less complete pathway reconstruction than in well-studied BIA branches. Major gaps include unresolved 3,4-DHBA formation, incomplete downstream tailoring characterization, and limited genomic and functional resources across Amaryllidoideae diversity.
Biotechnological applications for production	Advanced metabolic engineering and synthetic biology platforms for pathway reconstruction and production of IA/BIA intermediates and pharmaceutical alkaloids, especially in yeast and other heterologous systems.	Emerging metabolic engineering and transient plant-expression platforms for pathway reconstruction and production of AA intermediates and representative scaffold classes, especially in *Nicotiana benthamiana* and related heterologous systems.
Evolutionary interpretation	IA pathway diversification reflects broad taxonomic radiation and repeated recruitment or functional divergence of enzyme families such as CYP80, CYP719, methyltransferases, reductases, and tailoring enzymes across multiple eudicot lineages.	AA pathway diversification appears more lineage-restricted, with recent evidence showing functional divergence among closely related CYP96T homologs and downstream tailoring enzymes that redirect shared intermediates toward different AA scaffold types.

* The genes and enzymes listed are representative experimentally characterized or strongly supported modules discussed in this review, not an exhaustive inventory of all candidates. Detailed evidence, including species, substrate, method of discovery, and functional contribution, is provided in Supplementary Table S1.

**Table 2:** Omics-guided strategies for IA pathway discovery and functional gene prioritization across representative non-Amaryllidaceae plant systems.

Study	Species	Biological Question	Omics Approach	Strategy for Gene Discovery	Key Findings	Validation Approach/Tool
Desgagné-Penix et al., 2010 [41]	*Papaver somniferum* cell cultures	Which genes are associated with inducible alkaloid biosynthesis?	EST-based transcriptomics, proteomics (LC-MS/MS), and elicitor treatment	Elicitor-induced pathway activation, temporal expression profiling, and metabolite correlation	Identification of transcripts and proteins associated with sanguinarine biosynthesis; detection of most known pathway enzymes	Indirect/partial validation: proteomic detection by LC-MS/MS and correlation with metabolite accumulation; no direct recombinant enzyme assays
Marques et al., 2014 [42]	*Podophyllum hexandrum*/*P. peltatum*	Are alkaloid biosynthetic pathways present but previously undetected?	RNA-seq, metabolomics (UPLC-TOF-MS), and spatial metabolite imaging (MALDI-MS)	Homology-based pathway reconstruction combined with metabolite detection and spatial co-localization	Evidence supporting a previously unreported aporphine-related alkaloid pathway	Indirect validation: metabolite profiling and MALDI imaging; no direct enzymatic validation
Hagel et al., 2015 [37]	20 BIA-producing species	What is the diversity of biosynthetic enzymes across BIA-producing species?	Transcriptomics (Illumina and 454) and large-scale annotation	Homology-based mining, phylogenetic analysis, and expression filtering	Approximately 850 candidate genes across enzyme families, including CYPs and OMTs	Partial validation: enzymatic assays for selected *N*-methyltransferases
He et al., 2018 [55]	*Coptis chinensis*/*C. teeta*	Which genes are involved in BIA biosynthesis?	RNA-seq and pathway annotation (KEGG)	Homology mapping, differential expression analysis, and pathway reconstruction	Identification of more than 50 candidate genes associated with BIA pathways	Direct/partial validation: in vitro enzymatic assays for selected enzymes, including 6OMT and 7OMT
Pei et al., 2021 [53]	*Papaver somniferum*	How have BIA biosynthetic pathways evolved?	Genomics and multi-tissue transcriptomics	Comparative genomics, gene-family expansion analysis, and expression profiling	Expansion of gene families associated with BIA metabolism and pathway diversification, including signals of whole-genome duplication	Indirect validation: genomic and transcriptomic evidence; no direct enzymatic validation
Xu et al., 2021 [56]	*Corydalis yanhusuo*	How can BIA pathways be reconstructed without a reference genome?	Full-length transcriptomics (PacBio), RNA-seq, and metabolomics (UPLC-QTOF-MS)	Spatial expression filtering, metabolite correlation, and phylogenetic analysis	Prioritization of more than 100 candidate genes, including OMTs potentially involved in late BIA steps	Indirect validation: metabolite–gene correlation and phylogenetic inference
Li et al., 2022 [57]	*Stephania tetrandra*	How is tetrandrine biosynthesis organized?	Transcriptomics and metabolomics (LC-MS)	Metabolite-guided candidate selection and homology-based filtering	Identification of 42 candidate genes potentially involved in tetrandrine biosynthesis	Direct/partial validation: gene cloning, heterologous expression, and enzymatic assay for selected 6OMT candidates
Watkins et al., 2023 [58]	*Lophophora williamsii*	What is the biosynthetic pathway of mescaline?	Transcriptomics, metabolomics, and functional assays	Homology-based mining, candidate prioritization, and pathway reconstruction	Identification of key enzymes, including CYPs, decarboxylases, and OMTs, enabling mescaline pathway elucidation	Direct validation: gene cloning, heterologous expression in *E. coli*/yeast, and enzymatic assays

**Table 3:** Representative studies and strategies for mining functional genes involved in Amaryllidaceae alkaloid biosynthesis.

Study	Species	Biological Question/Target	Gene Discovery Strategy	Omics/Experimental Approach	Key Finding/Contribution	Validation Approach/Tool
Wang et al., 2013 [63]	*Lycoris aurea*	Which candidate genes are associated with AA biosynthesis?	De novo transcriptome mining	454 sequencing; tissue comparison; functional annotation	First transcriptomic resource for *L. aurea*; identification of candidate genes related to AA biosynthesis, including PAL, TYDC, OMTs, CYP450s, and NMTs	Candidate identification only; no direct enzymatic validation
Kilgore et al., 2014 [30]	*Narcissus* sp. *Aff. pseudonarcissus*	Which enzyme methylates norbelladine?	Co-expression-guided candidate selection	RNA-seq; HAYSTACK co-expression analysis; candidate cloning	Identification of N4OMT, catalyzing norbelladine 4′-*O*-methylation to form 4′-*O*-methylnorbelladine	Heterologous expression in *E. coli*; purified recombinant enzyme; kinetic assays
Kilgore et al., 2016a [31]	*Narcissus* sp. *Aff. pseudonarcissus*	Which enzyme catalyzes oxidative phenol coupling of 4′-*O*-methylnorbelladine?	Comparative transcriptomics + CYP candidate screening	Transcriptomics; homology-based CYP selection; biochemical assays	Identification of CYP96T1, mainly catalyzing para–para′ C–C phenol coupling, with minor para–ortho′ activity	Heterologous expression; enzymatic assays
Kilgore et al., 2016b [28]	*Narcissus* sp. *Aff. pseudonarcissus*	Which reductase participates in downstream AA biosynthesis and norbelladine formation?	Candidate selection from transcriptomic resources	Homology to SDR/tropinone reductase-like enzymes; recombinant protein assays	Identification of NR, a short-chain dehydrogenase/reductase active on noroxomaritidine and norcraugsodine-related substrates	Heterologous expression; purified enzyme assays
Singh and Desgagné-Penix, 2017 [64]	*Narcissus pseudonarcissus* ‘King Alfred’	Which transcriptomic and metabolomic components are associated with AA metabolism?	Integrated transcriptomics–metabolomics	Untargeted UPLC-QTOF-MS; Illumina RNA-seq; RT-qPCR	Generated a comprehensive transcriptome and identified proposed/known AA-related genes, including *TYDC*, *PAL*, *C4H*, *C3H*, *N4OMT*, *CYP96T1*, and *NR*	RT-qPCR validation and metabolite profiling; no new enzyme characterized
Singh et al., 2018 [26]	*Narcissus pseudonarcissus* ‘King Alfred’	Which enzyme catalyzes the first AA-specific committed step?	Pathway-gap targeting + homology to NCS-like enzymes	Transcriptome mining; candidate cloning; recombinant protein assays	Identification of NBS, involved in norbelladine formation from tyramine and 3,4-DHBA	Heterologous expression; enzymatic assays
Hotchandani et al., 2019 [65]	*Narcissus papyraceus*	How are AA biosynthetic genes regulated across tissues and development?	Developmental expression profiling	RNA-seq; cDNA library from 24 tissue/developmental samples; RT-qPCR; HPLC	Identification of 21 candidate AA biosynthetic genes; tissue- and development-dependent expression; lycorine reported as predominant AA	RT-qPCR + HPLC correlation; no new enzyme characterized
Wang et al., 2019 [67]	*Lycoris aurea*	Which TYDC contributes to tyramine supply for galanthamine biosynthesis?	Single-gene functional characterization	BLASTn-based candidate selection; cloning; heterologous expression; qRT-PCR; HPLC; MeJA/miRNA analysis	Characterization of LaTYDC1, catalyzing tyrosine-to-tyramine conversion; regulation by MeJA and miR396	*E. coli* expression; enzymatic assay; RLM-RACE confirmation of miRNA-mediated cleavage
Ferdausi et al., 2021 [66]	*Narcissus pseudonarcissus* cv. Carlton	How do field-derived basal plate and in vitro callus differ in secondary metabolism gene expression?	Differential transcriptomics	RNA-seq; reference-guided transcript analysis; GO/KEGG/Plant Reactome mapping	Identification of differentially expressed transcripts; late AA-related genes such as CYPs and OMTs were mainly upregulated in field basal plate, while precursor-related genes were more active in callus	Transcriptomic inference; no direct functional validation
Tousignant et al., 2022 [27]	*Leucojum aestivum*	Can AA biosynthetic genes be identified in *Leucojum*, and is NBS functional?	Integrated transcriptomics + functional validation	De novo transcriptomics; BLAST mining; phylogenetic analysis; heterologous expression; LC-MS/MS; subcellular localization	First transcriptome of *L. aestivum*; identification of ~50 AA-related genes; functional characterization of LaNBS	Recombinant enzyme assay by LC-MS/MS; cytosolic localization
Majhi et al., 2023 [29]	*Narcissus papyraceus* and *Leucojum aestivum*	Do NBS and NR act together in norbelladine formation?	Functional enzymology + protein interaction	Cloning; *E. coli* expression; enzymatic assays; molecular docking; yeast two-hybrid; split-luciferase in planta; subcellular localization	Demonstrated that NBS and NR cooperate in norbelladine biosynthesis and likely form a metabolon-like system	Recombinant enzyme assays; protein–protein interaction in yeast and planta; docking/modeling
Karimzadegan et al., 2024 [25]	*Leucojum aestivum*	Which phenylpropanoid enzymes contribute to 3,4-DHBA precursor formation?	Targeted functional characterization of precursor-pathway enzymes	Bioinformatics; cloning; yeast expression; in planta assays; subcellular localization; tissue-specific expression; metabolite profiling	Characterization of LaeC4H/CYP73A and LaeC3′H/CYP98A; evidence supporting phenylpropanoid contribution upstream of AA biosynthesis	Yeast assays; in planta validation; localization
Mehta et al., 2024 [32]	*Narcissus* cv. Tête-à-Tête; *N. papyraceus* for DESI-MS imaging	Where is AA biosynthesis active, and which enzymes form the galanthamine, lycorine, and haemanthamine branches?	Isotope-guided active-tissue discovery + long-read transcriptomics + transient pathway reconstruction	[^2^H]-4′-*O*-methylnorbelladine feeding; LC-MS; DESI-MS imaging; RT-qPCR; Illumina RNA-seq; PacBio Iso-Seq; *N. benthamiana* transient expression	Active biosynthesis was localized to young leaf bases; identification of NtCYP96T5 for ortho–para′ coupling, NtCYP96T6 for para–ortho′ coupling, and core enzymes for haemanthamine and galanthamine biosynthesis, including NtSDR1/2, NtCYP71DW1, NtODD2, NtOMT1, NtNMT1, and NtAKR1	Stable-isotope precursor feeding; LC-MS/MS; DESI-MS imaging; *N. benthamiana* transient expression; yeast microsomes; site-directed mutagenesis; authentic-standard comparison
Liu et al., 2024 [33]	*Lycoris aurea*	How do CYP96T homologues control para–para′ and para–ortho′ oxidative coupling?	Full-length transcriptomics + CYP96T functional diversification analysis	PacBio SMRT full-length transcriptome; Illumina RNA-seq across tissues and MeJA treatments; homology mining; CYP96T cloning; subcellular localization; *N. benthamiana* transient expression	Characterization of four LauCYP96T enzymes; LauCYP96T1 and LauCYP96T1-like-2 mainly produced para–para′ products, whereas LauCYP96T1-like-1 and LauCYP96T1-like-3 favored para–ortho′ products	ER localization; LC-MS with standards; *N. benthamiana* transient expression; chimera/mutagenesis assays; homology modeling and docking
Transcriptomic resources compiled by Lamichhane et al., 2025 [34]	Multiple Amaryllidaceae/Amaryllidoideae species, including *Crinum*, *Galanthus*, *Hippeastrum*, *Leucojum*, *Lycoris*, *Narcissus*, *Zephyranthes*, and related genera	What transcriptomic resources are available for AA pathway mining across Amaryllidaceae?	Comparative survey of publicly available transcriptomic resources	Compilation of transcriptome assemblies and accession numbers from published studies and public databases	Shows that transcriptomic resources for Amaryllidaceae are broader than previously stated, but unevenly distributed across genera and not always linked to functional validation	Resource-level evidence; no direct enzyme validation from the table itself
Lamichhane et al., 2025 [34]	*Leucojum aestivum*	Can AA biosynthesis be reconstructed from precursors in planta?	Pathway reconstruction + synthetic biology	Transcriptome-derived candidate selection; phylogeny; docking; subcellular localization; *Agrobacterium*-mediated transient expression in *Nicotiana benthamiana*	Reconstitution of foundational and branching AA steps; LaNBS + LaNRII produced norbelladine, LaN4′OMT produced 4′-*O*-methylnorbelladine, and LaCYP96T1/2 generated phenol-coupled products associated with lycorine-, galanthamine-, and crinine/haemanthamine-type alkaloids	In planta transient expression; LC-MS/MS; in vitro assays; microsome assays; subcellular localization; docking

**Table 4:** Candidate routes and evidence strength for 3,4-dihydroxybenzaldehyde (3,4-DHBA) formation in Amaryllidaceae alkaloid biosynthesis.

Candidate Route	Proposed Key Enzymes/Steps	Species Tested	Supporting Evidence	Current Evidence Strength	Main Limitation
Phenylpropanoid route through trans-cinnamic acid and *p*-coumaric acid	PAL → C4H/CYP73A → *p*-coumaric acid → downstream hydroxylation/shortening steps	*Leucojum aestivum*, *Lycoris* spp.	LaeC4H converts trans-cinnamic acid to *p*-coumaric acid; phenylpropanoid-related transcripts and metabolites are detected in AA-producing tissues	Strong for LaeC4H-mediated trans-cinnamate → *p*-coumaric acid; weak for downstream conversion to 3,4-DHBA.	Does not explain the complete conversion to 3,4-DHBA; downstream enzymes remain unidentified
C3′H/CYP98A-dependent route through *p*-coumaroyl shikimate	4CL/HCT → *p*-coumaroyl shikimate → LaeC3′H/CYP98A → caffeoyl shikimate → CSE → caffeic acid → unknown conversion to 3,4-DHBA	*Leucojum aestivum*	LaeC3′H catalyzes 3-hydroxylation of *p*-coumaroyl shikimate	Strong for *p*-coumaroyl shikimate → caffeoyl shikimate; weak for direct caffeic acid or 3,4-DHBA formation.	LaeC3′H did not hydroxylate free *p*-coumaric acid or 4-HBA (4-hydroxybenzaldehyde), and no DHBA was detected from these substrates.
Direct hydroxylation of free *p*-coumaric acid to caffeic acid	APX/C3H or related *p*-coumarate 3-hydroxylase	*Leucojum aestivum*	LaeAPX/C3H consumes *p*-coumaric acid	Weak or unsupported in *L. aestivum*.	Although *p*-coumaric acid was consumed, caffeic acid accumulation was higher in the negative control, indicating that caffeic acid production was not enzyme-dependent.
4-Hydroxybenzaldehyde hydroxylation route	4-hydroxybenzaldehyde → 3,4-DHBA via putative hydroxylase	*Leucojum aestivum*	4-HBA has been proposed as a possible intermediate	Hypothetical; not supported for LaeC3′H or LaeAPX/C3H in *L. aestivum*.	LaeC3′H did not hydroxylate 4-HBA, and LaeAPX/C3H did not show significant enzyme-dependent 4-HBA consumption or DHBA production.
Ferulic acid/ferulate-derived route	Caffeic acid → ferulic acid or related phenylpropanoid intermediates → aldehyde derivatives	AA-producing species, mostly inferred	Ferulic acid and related intermediates are detected in some metabolite profiles	Weak/indirect	No characterized enzyme connects ferulate-derived intermediates to 3,4-DHBA in AA biosynthesis
Retrobiosynthetic/chemical logic route	Structural inference from norbelladine and downstream AA skeletons	Multiple Amaryllidaceae species	Feeding and radiolabeling studies support phenylalanine contribution to the aldehyde moiety	Moderate for phenylalanine contribution, weak for enzymatic sequence.	Supports precursor origin but does not identify the enzymatic sequence

Evidence summarized primarily from Karimzadegan et al. [25] and previous biosynthetic interpretations of AA precursor origin [24,70].

**Table 5:** Qualitative comparison of biotechnological production platforms for IA and AA compounds.

Production Platform	Main Advantages	Main Limitations	Current Relevance for IAs and AAs
Plant extraction	Established source of natural alkaloids; no pathway reconstruction required	Low or variable yield, slow growth, environmental dependence, sustainability concerns, high downstream extraction cost	Still important for many AAs such as galanthamine; also, historically important for IAs such as morphine
In vitro callus/cell cultures	Controlled growth conditions; compatible with elicitors and precursor feeding; useful for studying regulation	Moderate or unstable yields; strong dependence on genotype, tissue type, and culture conditions	Useful for AA and IA pathway studies, but not always sufficient for scalable production
Microbial synthesis	Scalable, standardized, engineerable; suitable for pathway optimization and new-to-nature derivatives	Requires complete pathway knowledge, enzyme compatibility, precursor balancing, toxicity management, and high initial engineering effort	More advanced for BIAs/IAs; still emerging for AAs because several pathway steps remain unresolved
Transient plant expression	Rapid functional testing in planta; supports multi-enzyme reconstruction; useful for CYPs and plant-specific reactions	Generally, a discovery/reconstruction platform rather than a mature industrial production system	Recently powerful for AAs through *N. benthamiana* reconstruction; also useful as a bridge toward stable chassis development

## Data Availability

Data are contained within the article and Supplementary Materials.

## Supplementary Materials

The following supporting information can be downloaded at: https://www.mdpi.com/article/10.3390/plants15172553/s1, Table S1. Representative characterized enzymes and regulatory factors involved in IA and AA biosynthesis and the methodological evidence supporting their functional assignment. References [75,76,77,78,79,80,81] are cited in the supplementary materials.
