# Supplementary material for: Amaryllidaceae Alkaloids and Isoquinoline Alkaloids: A Perspective on Historical Approaches to Pathway Elucidation
Source: Plants (Basel). 2026 Aug 22;15(17):2553. doi: 10.3390/plants15172553 (PMC13567729; doi:10.3390/plants15172553)
Supplement: Supplementary file 1 [file plants-15-02553-s001.zip › plants-3906334-supplementary.pdf]

plants

MDPI

Review

Amaryllidaceae Alkaloids and Isoquinoline Alkaloids: A Perspective on Historical Approaches to Pathway Elucidation

Mateo Pena-Morales [1], Jaime David Vega-Paez [2], Natalie Cortes [3], Edison Osorio [4], Paola A. Caicedo [1], Alvaro Barrera-Ocampo [1], Oscar Alvarez [5], Andres Fernando Gonzales Barrios [5] and Maria Francisca Villegas-Torres [2,*]

1 Grupo Natura, Facultad Barberi de Ingenieria, Diseno y Ciencias Aplicadas, Universidad ICESI, Cali 760031, Colombia; mapena@icesi.edu.co (M.P.-M.); pacaicedo@icesi.edu.co (P.A.C.); aabarrera@icesi.edu.co (A.B.-O.)
2 Centro de Investigaciones Microbiologicas (CIMIC), Department of Biological Sciences, Universidad de los Andes, Bogota 111711, Colombia; jd.vega1754@uniandes.edu.co
3 Facultad de Ciencias Naturales y Matematicas, Universidad de Ibague, Carrera, 22, Calle 67, Ibague 730002, Colombia; charlotte08@gmail.com
4 Grupo de Ciencias Basicas Aplicadas (CBATA), Departamento de Ciencias Basicas y Areas Comunes, Tecnologico de Antioquia-Institucion Universitaria, Medellin 050034, Colombia; edison.osorio@tdea.edu.co
5 Grupo de Diseno de Productos y Procesos (GDPP), Department of Chemical and Food Engineering, Universidad de los Andes, Bogota 111711, Colombia; oalvarez@uniandes.edu.co (O.A.); andgonza@uniandes.edu.co (A.F.G.B.)
* Correspondence: m.f.villegastorres@uniandes.edu.co

 https://doi.org/10.3390/plants15172553

Supplementary Table S1. Representative characterized enzymes and regulatory factors involved in IA and AA biosynthesis and the methodological evidence supporting their functional assignment.

Alkaloid group	Enzyme / gene	Year	Species	Study / research group	Substrate or target	Product / reaction	Discovery or validation method	Contribution to pathway elucidation
IAs / BIAs	NCS, norcoclaurine synthase	2002	*Thalictrum flavum* ssp. *glaucum*	Samanani & Facchini [13]	Dopamine + 4-hydroxyphenylacetaldehyde	*(S)*-norcoclaurine	Enzyme purification from cell suspension cultures; radiolabeled enzyme assay; chromatographic purification; kinetic characterization	Established NCS as the first committed enzyme of benzylisoquinoline alkaloid biosynthesis and clarified formation of the central entry intermediate *(S)*-norcoclaurine.
IAs / aporphine branch	CYP80G2	2008	*Coptis japonica*	Ikezawa, Iwasa & Sato [36]	*(S)*-reticuline	*(S)*-corytuberine	EST-library mining; cDNA isolation; heterologous expression in yeast; microsomal enzyme assays; LC-MS/MS and LC-NMR product identification	Identified a cytochrome P450 catalyzing intramolecular C–C phenol coupling, supporting CYP80G-mediated aporphine-type scaffold formation.

IAs / morphinan branch	STORR, P450–oxidoreductase fusion protein	2015	*Papaver somniferum*	Winzer et al. [14]	(*S*)-reticuline; 1,2-dehydroreticuline	(*S*)-reticuline → 1,2-dehydroreticuline → (*R*)-reticuline	Candidate-gene analysis; mutant genetic analysis; metabolite profiling; heterologous expression in *Saccharomyces cerevisiae*; enzyme assays; proteomic confirmation of the fusion protein	Resolved the gateway epimerization step directing metabolites from (*S*)-reticuline into the morphinan branch.
IAs / morphinan branch	NISO / PR10-3, neopinone isomerase	2019	*Papaver somniferum*	Dastmalchi et al. [17]	Neopinone; neomorphinone	Neopinone ßà codeinone; neomorphinone ßà morphinone	Latex proteomics and transcriptomics; PR10/MLP candidate screening; recombinant protein assays coupled with COR; time-course assays; engineered yeast assays; transcript suppression in opium poppy	Demonstrated that a presumed spontaneous isomerization in late morphinan biosynthesis is enzymatically catalyzed, improving codeine and morphine pathway reconstruction.

AAs	N4OMT / NpN4OMT	2014	*Narcissus sp. Aff. pseudo-narcissus*	Kilgore et al. [30]	Norbelladine	4′-*O*-methylnorbelladine	De novo transcriptome assembly; BLAST identification of O-methyltransferase candidates; HAYSTACK co-expression analysis; heterologous expression in *E. coli*; affinity purification; enzymatic assay	First molecularly characterized committed AA biosynthetic gene; validated norbelladine 4′-*O*-methylation toward the universal AA intermediate 4′-*O*-methylnorbelladine.
AAs	CYP96T1	2016	*Narcissus sp. Aff. pseudo-narcissus*	Kilgore et al. [31]	4′-*O*-methylnorbelladine	Mainly para–para′ C–C phenol-coupled noroxomaritidine products; minor para–ortho′ N-demethylnarwedine	Comparative transcriptomics of *Narcissus*, *Galanthus* sp., and *Galanthus elwesii*; co-expression with N4OMT; heterologous expression; biochemical characterization	Identified the first monocot phenol-coupling CYP in AA biosynthesis and validated the para–para′ oxidative coupling branch leading toward haemanthamine/crinine-type scaffolds.

AAs	NR, noroxomaritidine reductase	2016	*Narcissus sp. Aff. pseudonarcissus*	Kilgore et al. [28]	Noroxomaritidine; norcraugsodine with lower activity	Oxomaritinamine from noroxomaritidine; norbelladine from norcraugsodine with much lower specific activity	Co-expression analysis with N4OMT; cloning of an SDR candidate; heterologous expression in *E. coli*; biochemical assays; LC-MS/MS; X-ray crystallography	Identified an SDR involved in reductive tailoring of AA intermediates and provided early evidence that NR-type enzymes can also participate in norbelladine formation.
AAs	NBS / NpNBS	2018	*Narcissus pseudonarcissus*	Singh et al. [26]	Tyramine + 3,4-DHBA	Norbelladine	Transcriptome mining for NCS orthologs; cloning; heterologous expression in *E. coli*; recombinant enzyme assay; HR-MS/MS product identification	Identified norbelladine synthase as a PR10/Bet v1-like enzyme catalyzing the first committed reaction proposed for AA biosynthesis.

AAs	NBS + NR	2023	*Narcissus papyraceus* and *Leucojum aestivum*	Majhi et al. [29]	Tyramine + 3,4-DHBA; norcraugsodine	Norcraugsodine → norbelladine	Transcriptome mining; cloning; recombinant expression in *E. coli*; enzyme assays with enzymes alone and combined; homology modeling/docking; yeast two-hybrid; in planta interaction assays; subcellular localization; co-expression in yeast	Established a two-step route for norbelladine formation involving NBS-mediated imine formation and NR-mediated reduction; supported physical and functional NBS–NR cooperation.
AAs / precursor supply	LaeC4H / CYP73A	2024	*Leucojum aestivum*	Karimzadegan et al. [25]	trans-cinnamate	*p*-coumaric acid	Bioinformatics; cloning; heterologous expression in yeast; in planta assays in *Nicotiana benthamiana*; subcellular localization; tissue-specific expression; metabolite profiling	Functionally characterized an upstream phenylpropanoid enzyme contributing to the proposed route toward the 3,4-DHBA precursor, while confirming that 3,4-DHBA formation itself remains unresolved.

AAs / precursor supply	LaeC3′H / CYP98A	2024	*Leucojum aestivum*	Karimzadegan et al. [25]	*p*-coumaroyl shikimate	Caffeoyl shikimate	Yeast expression; in planta assays; subcellular localization; tissue-specific expression; metabolite profiling	Showed that LaeC3′H hydroxylates *p*-coumaroyl shikimate rather than free *p*-coumaric acid or 4-hydroxybenzaldehyde, refining the working hypothesis for 3,4-DHBA precursor formation.
AAs / oxidative coupling	LauCYP96T1 and LauCYP96T1-like homologs	2024	*Lycoris aurea*	Liu et al. [33]	4′-*O*-methylnorbelladine	Para–para′ and para–ortho′ oxidative coupling products	PacBio full-length transcriptome; CYP candidate mining; cloning of four LauCYP96T homologs; ER localization; functional assays; homology modeling and docking	Demonstrated functional diversity and inverted regioselectivity among closely related CYP96T homologs; showed that CYP96T numbering/function cannot be generalized across species.

AAs / active-tissue discovery and oxidative coupling	NtCYP96T5, NtCYP96T6, NtSDR1	2024 [32]	*Narcissus* cv. Tête-à-Tête	Mehta et al.	4′-*O*-methylnorbelladine	NtCYP96T6 catalyzes para–ortho′ coupling; NtCYP96T5 together with NtSDR1 produces norpluviine in the ortho–para′ branch	Isotope-guided identification of active leaf-base tissue; RNA-seq and PacBio Iso-Seq; P450 candidate mining; transient expression in *Nicotiana benthamiana*; LC-MS/MS; yeast microsomes; site-directed mutagenesis	Identified CYP96T homologs mediating alternative oxidative coupling modes, providing access to galanthamine- and lycorine-related branches.
AAs / haemanthamine branch	NtSDR2, NtCYP71DW1, NtODD2, NtOMT1	2024	*Narcissus* cv. Tête-à-Tête	Mehta et al. [32]	Noroxomaritidine-derived intermediates downstream of 4′-*O*-methylnorbelladine	NtSDR2 reduces noroxomaritidine to normaritidine; NtCYP71DW1 forms the methylenedioxy bridge to vittatine; NtODD2 hydroxylates vittatine; NtOMT1 methylates 11-hydroxyvittatine to haemanthamine	Chemical logic and co-expression analysis; active-tissue RNA-seq; transient expression in *Nicotiana benthamiana*; LC-MS/MS comparison with standards	Reconstituted the core haemanthamine pathway in a heterologous plant system.

AAs / galanthamine branch	NtCYP96T6, NtNMT1, NtAKR1	2024	*Narcissus* cv. Tête-à-Tête	Mehta et al. [32]	4′-*O*-methylnorbelladine and demethylnarwedine/narwedine intermediates	NtCYP96T6 produces demethylnarwedine; NtNMT1 methylates demethylnarwedine to narwedine; NtAKR1 reduces narwedine to galanthamine	Chemical logic and co-expression analysis; active-tissue RNA-seq; transient expression in *Nicotiana benthamiana*; LC-MS/MS; pathway reconstitution	Identified a core gene set for galanthamine biosynthesis in daffodil.
AAs / regulatory factor	LaMYC2	2024	*Lycoris aurea*	Zhou et al. [75]	LaTYDC promoter; JA signaling module	Positive regulation of lycorine biosynthesis; activation of LaTYDC and LaPAL expression	Comparative transcriptomics under MeJA treatment; TF correlation with lycorine content; cloning; nuclear localization; LaJAZ interaction; transient overexpression in petals; promoter binding assay	Provided functional evidence that jasmonate-responsive transcriptional regulation contributes to AA biosynthesis through LaMYC2-mediated activation of precursor-supply genes.

AAs / pathway reconstruction	LaNBS, LaNRII, LaN4′OMT, LaCYP96T1, LaCYP96T2	2025	*Leucojum aestivum*	Lamichhane et al. [34]	Tyramine + 3,4-DHBA; norbelladine; 4′-*O*-methylnorbelladine	Norbelladine; 4′-*O*-methylnorbelladine; lycorine-, galanthamine-, and crinine/haemanthamine-related products	Candidate selection from *L. aestivum* transcriptome; cloning; Agrobacterium-mediated transient expression in *Nicotiana benthamiana*; co-agroinfiltration; HPLC-MS/MS; in vitro assays; modeling/docking	Reconstituted key AA foundation steps and tested CYP96T-mediated scaffold diversification in planta, moving AA research from candidate validation toward pathway reconstruction.
IAs / BIAs / reticuline pathway	6OMT and 4′OMT	2010	*Coptis japonica*	Morishige et al. [76]	6OMT: *(S)*-norcoclaurine; 4′OMT: 3′-hydroxy-*N*-methylcoclaurine; chimeric OMT substrates	6OMT catalyzes 6-*O*-methylation toward *(S)*-coclaurine; 4′OMT catalyzes 4′-*O*-methylation toward *(S)*-reticuline; chimeric OMT analysis showed altered substrate specificity	Chimeric enzyme construction using 6OMT, 4′OMT, and SMT domains; heterologous expression in *E. coli*; HPLC and LC-MS activity assays; substrate-specificity testing	Supported the functional importance of OMT regioselectivity in BIA biosynthesis and showed that N-terminal regions contribute to substrate recognition and reaction specificity in IA-related OMTs.

IAs / BIAs / reticuline pathway	CNMT, coclaurine *N*-methyltransferase	2002	*Coptis japonica*	Choi et al. [77]	Coclaurine	*N*-methylcoclaurine	Purification-informed cDNA cloning; RACE-based full-length cDNA isolation; heterologous expression in *E. coli*; recombinant enzyme purification; enzymatic characterization	Established CNMT as the *N*-methyltransferase step linking coclaurine to *N*-methylcoclaurine in the route toward *(S)*-reticuline and showed that CNMT has relatively broad substrate specificity.
IAs / benzophenanthridine and protoberberine-related branches	BBE, berberine bridge enzyme / reticuline oxidase	1995	*Eschscholzia californica*	Dittrich & Kutchan [78]	*(S)*-reticuline	*(S)*-scoulerine through oxidative berberine-bridge formation	Molecular cloning; heterologous expression in *Saccharomyces cerevisiae*; functional enzyme expression; induction analysis	Identified BBE as the FAD-dependent oxidase catalyzing berberine bridge formation from *(S)*-reticuline, establishing a key branch-forming reaction toward protoberberine and benzophenanthridine alkaloids.

IAs / morphinan branch	SalSyn / CYP719B1, salutaridine synthase	2009	*Papaver somniferum*	Gesell et al. [15].	*(R)*-reticuline	Salutaridine	Candidate CYP identification; molecular cloning; heterologous expression; microsomal enzyme assays; product identification	Identified the CYP719B1-mediated intramolecular C–C phenol-coupling step that commits *(R)*-reticuline to the morphinan branch through salutaridine formation.
IAs / morphinan branch	SalR, salutaridine reductase	1993 / 2010	*Papaver somniferum*	Gerardy & Zenk; Higashi et al. [79, 80]	Salutaridine + NADPH	Salutaridinol	Enzyme purification and biochemical characterization from *P. somniferum* cultures/tissues; later structural characterization by X-ray crystallography with NADPH and substrate-docking analysis	Established SalR as the stereoselective SDR enzyme reducing salutaridine to salutaridinol, linking salutaridine formation to salutaridinol 7-*O*-acetate and thebaine biosynthesis.
IAs / morphinan branch	SalAT, salutaridinol 7-*O*-acetyltransferase	1995 / 2001	*Papaver somniferum*	Lenz & Zenk; Grothe et al. [54, 16]	Salutaridinol + acetyl-CoA	Salutaridinol 7-*O*-acetate	Enzyme purification and biochemical characterization; later molecular cloning, heterologous expression, and functional characterization	Established the acetylation step that produces salutaridinol 7-*O*-acetate, the immediate precursor to thebaine formation in morphinan biosynthesis.

IAs / morphinan branch	THS, thebaine synthase	2018	*Papaver somniferum*	Chen et al. [20]	*(7S)*-salutaridinol 7-*O*-acetate	Thebaine	Enzyme detection and purification from latex; protein identification; functional enzyme assays; genomic/gene-cluster analysis; engineered yeast validation	Demonstrated that the final step to thebaine is catalyzed by a PR10 protein rather than occurring only by spontaneous allylic elimination, improving morphinan pathway reconstruction and yeast-based thebaine production.
IAs / morphinan branch	T6ODM, thebaine 6-*O*-demethylase	2010	*Papaver somniferum*	Hagel & Facchini [18]	Thebaine	Neopinone / codeinone-route intermediates through 6-*O*-demethylation	Candidate dioxygenase identification; molecular cloning; recombinant enzyme assays; biochemical characterization of 2-oxoglutarate/Fe(II)-dependent dioxygenase activity	Identified T6ODM as a key *O*-demethylase in late morphinan biosynthesis, clarifying the conversion of thebaine toward codeine and morphine.

IAs / morphinan branch	CODM, codeine *O*-demethylase	2010	*Papaver somniferum*	Hagel & Facchini [18]	Codeine; also oripavine-related substrates depending on branch context	Morphine; morphinone-related products	Candidate dioxygenase identification; molecular cloning; recombinant enzyme assays; biochemical characterization of 2-oxoglutarate/Fe(II)-dependent dioxygenase activity	Identified CODM as the enzyme catalyzing the final *O*-demethylation of codeine to morphine and contributed to resolving the bifurcated late morphinan pathway.
IAs / morphinan branch	COR, codeinone reductase	1999	*Papaver somniferum*	Unterlinner et al. [19]	Codeinone; morphinone	Codeine; morphine	Molecular cloning; heterologous expression; recombinant enzyme activity assays	Identified COR as the reductase catalyzing the penultimate reduction of codeinone to codeine and supporting morphinone-to-morphine conversion in the alternative late morphinan branch.

IAs / regulation and metabolic modulation	Multiple BIA genes including T6ODM, CODM, and COR	2019	*Papaver somniferum* cv. Sujata	Ray et al. [81]	Endophyte-regulated BIA pathway genes and alkaloid intermediates	Increased morphine and thebaine accumulation; enhanced expression of morphinan-related genes including COR	Endophytic consortium inoculation; qRT-PCR of BIA biosynthetic genes; HPLC alkaloid profiling; physiological measurements	Not a primary enzyme-characterization study, but useful as regulatory/modulatory evidence showing that endophyte consortia can alter BIA gene expression and morphinan alkaloid accumulation in planta.
